# *Polygala tenuifolia* and *Acorus tatarinowii* in the treatment of Alzheimer’s disease: a systematic review and meta-analysis

**DOI:** 10.3389/fphar.2023.1268000

**Published:** 2024-01-12

**Authors:** Yuchen Zhang, Jinzhou Tian, Jingnian Ni, Mingqing Wei, Ting Li, Jing Shi

**Affiliations:** ^1^ Dongzhimen Hospital, Beijing University of Chinese Medicine, Beijing, China; ^2^ Department of Neurology, Dongzhimen Hospital, Beijing University of Chinese Medicine, Beijing, China

**Keywords:** *Acorus tatarinowii*, Alzheimer’s disease, clinical application, herb combinations, *Polygala tenuifolia*, traditional Chinese medicine, randomized controlled trials

## Abstract

**Background:** The complexity of Chinese medicine treatment for Alzheimer’s disease (AD) utilizing a multi-herb therapy makes the evidence in current studies insufficient. Herb pairs are the most fundamental form of multi-herb formulae. Among the Chinese herbal formulas for AD treatment, *Polygala tenuifolia* (PT) and *Acorus tatarinowii* (AT) appeared as the most commonly used herbal pairs in combination.

**Objective:** The aim of this study is to evaluate the clinical efficacy and safety of the combination of PT and AT in the treatment of AD.

**Methods:** We systematically searched and screened randomized controlled trials of pairing PT and AT for the treatment of AD patients in eight databases with a search deadline of June 26, 2023. Authors, year of publication, title, and basic information such as subject characteristics (age, sex, and race), course of disease, control interventions, dose, and treatment duration were extracted from the screened studies. Primary outcomes assessed included mini-mental state examination (MMSE), activities of daily living (ADL), and AD assessment scale-cognitive subscale (ADAS-cog), while secondary outcomes included efficiency and adverse events. The quality of the included studies was assessed using the Cochrane risk of bias tool. The mean difference with 95% confidence intervals (MD [95% CI]) and risk ratio (RR) was selected as the effect size, and the data were analyzed and evaluated using RevMan 5.4 and Stata 16.

**Results:** A total of sixteen eligible and relevant studies involving 1103 AD participants were included. The combination of PT and AT plus conventional drugs was superior to single conventional drugs in MMSE [MD = 2.57, 95%CI: (1.44, 3.69); *p* < 0.00001; *I* ^*2*^ = 86%], ADL [MD = −3.19, 95%CI: (−4.29, −2.09); *p* < 0.00001; *I* ^*2*^ = 0%], and ADAS-cog scores [MD = −2.09, 95%CI: (−3.07, −1.10); *p* < 0.0001; *I* ^*2*^ = 0%]. The combination of PT and AT plus conventional drugs had a significantly more favorable benefit in clinical effectiveness [RR = 1.27, 95%CI: (1.12, 1.44); *p* = 0.0002; *I* ^*2*^ = 0%]. Adverse events were not increased with the combination of PT and AT plus conventional drugs compared to conventional drugs [RR = 0.65, 95%CI: (0.35, 1.19); *p* = 0.16; *I* ^*2*^ = 0%]. The experimental group treated with the combination of PT and AT alone for AD was comparable in MMSE, ADL, and ADAS-cog scores compared with the control group treated with single conventional drugs.

**Conclusion:** Compared to single conventional drugs, the combination of PT and AT may be used as an alternative therapy to improve global cognition and functioning in AD, and the combination of PT and AT as adjunctive therapy appears to produce a better therapeutic response to AD in terms of efficacy without increasing the risk of adverse events. However, the very low to low quality of available evidence limits confidence in the findings.

**Systematic Review Registration:** https://www.crd.york.ac.uk/prospero/, identifier CRD42023444156.

## Introduction

Alzheimer’s disease (AD) is a progressive neurodegenerative disorder characterized by memory loss, insidious episodes of cognitive deficits, and multiple dysfunctions such as aphasia, apraxia, agnosia, or executive decline, either individually or in combination (Dafre and Wasnik, 2023). The increasing aging of the global population and the increasing prevalence of AD have transformed AD into a major global medical, social, and economic dilemma (Passeri et al., 2022a). Currently, 50 million individuals are living with dementia globally, and this figure is projected to escalate to 152 million by 2050 (Scheltens et al., 2021).

Existing approved and marketed therapeutic strategies for AD include drugs such as donepezil, galantamine, carbapenems, and memantine, which belong to two families of anticholinesterase inhibitors and antiglutamatergic drugs (Balázs et al., 2021; Doroszkiewicz and Mroczko, 2022). These medications are used to limit disease progression, stabilize cognitive function, and attempt to improve the quality of life for patients with AD (Se Thoe et al., 2021; Srivastava et al., 2021). Nonetheless, current treatment options are only able to improve the clinical symptoms of AD patients for a limited period, and curative treatment is still not available (Plascencia-Villa and Perry, 2021). Studies have shown that none of the medications currently approved by the Food and Drug Administration (FDA) for AD treatment have positive effects on neuronal damage and brain atrophy or even progressive deterioration of cognition (Vaz and Silvestre, 2020). It is also worth noting that the latest monoclonal antibody approved by the FDA as an immunotherapy, aducanumab, has shown very limited improvement in cognitive function in AD, and its effectiveness remains highly controversial to date (Syed, 2020). Therefore, the search for new and effective treatments is a necessity.

Over thousands of years, the advantages of the diagnostic and therapeutic experience accumulated by Chinese medicine are extremely significant and have demonstrated an important role in the multi-target and multi-pathway treatment of AD (Deng et al., 2022; Ding et al., 2022; Arora et al., 2023). *Polygala tenuifolia* (PT) is a well-known traditional Chinese medicine, which has neuroprotective properties for treating amnesia and improving mental ability, with mechanisms of action involving anti-inflammatory, autophagic, anti-apoptotic, and anti-oxidative stress (Wang et al., 2021; Zhang et al., 2023). *Acorus calamus* (AT) is a traditional natural medicinal plant in the empirical system of Chinese medicine, and modern pharmacological studies have provided evidence of the pharmacological effects of its bioactive constituents, which can significantly improve brain diseases and neurological disorders such as Alzheimer’s disease (Zhang et al., 2022; Xu et al., 2023; Wang et al., 2023). The beneficial effects of the combination of PT and AT in ameliorating cognitive symptoms of AD through mechanisms such as the inhibition of inflammatory factors and modulation of intestinal flora have been demonstrated in preclinical studies (Xiong et al., 2022a; Jiao et al., 2022). Kaixin San (KXS), recorded in Sun Simiao’s “*Prescriptions for Emergencies*” in the Tang Dynasty, was used to treat dementia which has significant efficacy (Bo et al., 2022). In addition, the combination of PT and AT is frequently used in all medications and is a core prescription for the treatment of dementia (Yi et al., 2018).

Modern pharmacological studies have shown that the active constituents of PT and AT and their therapeutic mechanisms are complex. Polygala saponins are the major bioactive constituents of PT and have therapeutic potential in various neurological disorders. PTBP-1-3, a heteropolysaccharide isolated from PT, has an extremely potent inhibitory effect on neuroinflammation and may be one of the bioactive components in PT to improve cognitive function. It has been shown that tenuifolin, isolated from PT roots, has a significant improvement in cognitive function, preventing apoptosis, loss of mitochondrial membrane potential, and activation of caspases-3 in SH-SY5Y cells. It was found that PT may inhibit the accumulation of hyperphosphorylated tau through the 26S proteasome pathway to improve tau binding to microtubules, thereby improving cognitive performance. SCP-oil is the main active component of AT. SCP-oil promotes neuroprotection by reducing the activation of NLRP3 inflammatory vesicles through the inhibition of the NF-κB signaling pathway. ATP50-3, an active component of crude polysaccharide from AT, exerts anti-neuroinflammatory and neuroprotective effects by modulating the TLR4-mediated MyD88/NF-κB and PI3K/Akt signaling pathways.

In recent decades, TCM research has predominantly concentrated on cultivating potential candidates from Chinese medicinal herbs, with insufficient attention given to the judicious application of these traditional herbs. While multi-herb therapy stands as a pivotal characteristic of TCM, the contemporary endeavor to modernize this conventional wisdom encounters formidable challenges owing to its staggering complexity (Wang et al., 2012). Herb pairs, constituting the fundamental and simplest form of multi-herb formulations, serve as a central representative of Chinese herbal compatibility. TCM has multiple targets, and the efficacy and value of the combination of PT and AT, as a herb pair with unique advantages of TCM, have not yet been evaluated for its practical clinical application in the treatment of AD. We carried out a comprehensive systematic review and meta-analysis to explore the clinical efficacy and safety of combining PT and AT in the treatment of AD. This is the first systematic review and meta-analysis aimed to evaluate the global cognition and functioning effect and safety of the combination of PT and AT used alone or as adjunctive therapies in randomized controlled trials (RCTs) for the treatment of AD.

## Methods

### Protocol and registration

This study was performed following the Preferred Reporting Items for Systematic Reviews and Meta-Analysis guidelines and was registered in the International Prospective Register of Systematic Reviews (Number CRD42023444156).

### Inclusion and exclusion criteria

Inclusion criteria: Types of studies: RCTs were included irrespective of blinding and language. Participants: Patients with AD, no restrictions on case origin, age, sex, or disease duration. Clear diagnostic criteria are proposed; Western medical diagnostic criteria meet one of the following diagnostic criteria: a) NIA-AA, b) NINCDS-ADRDA, c) DSM-Ⅴ, d) IWG-2, and e) ICD-10. A clear TCM syndrome type is proposed; TCM diagnostic criteria meet one of the following diagnostic criteria: a) Guidance principle of clinical study on new drugs of traditional Chinese medicine and b) Criteria for diagnosis, diagnostic typing, and efficacy assessment of senile dementia. Types of interventions: The intervention in the experimental group was any form of formula that included all types of PT and AT used in combination, either alone or in combination with conventional AD treatment with drugs. There were no restrictions on dosage, frequency of use, or formulations. Treatment duration was ≥ 12 weeks. Comparison: Interventions in the control group included no treatment, placebo, or conventional drugs for AD with the unrestricted mode of administration and dosage.

Exclusion criteria: Duplicate studies including reviews, guidelines, letters, conference abstracts, commentaries, case reports, and animal and cellular experiments; medical history of neurological, psychiatric, or other systemic disorders that may have an impact on cognitive function (e.g., depression, stroke, vascular dementia (VaD), and mild cognitive impairment); interventions combined with other TCM treatments such as acupuncture, moxibustion, auricular acupuncture, and physiotherapy; lack of quantitative data on outcome indicators and research with incomplete or unavailable data; and failure to provide study data for mean and SD or SE or CI.

### Database search

Two authors (JZT and MQW) conducted independent searches of PubMed, Embase, Cochrane, the Web of Science, China National Knowledge Infrastructure (CNKI), the Wanfang Database, SINOMED, and the China Science and Technology Journal Database to screen and collect relevant studies published up to June 26, 2023.

### Search strategy

The terms and search strategy were as follows: “(Alzheimer disease OR Alzheimer* OR dementia OR AD OR cogniti*) AND (Polygala OR Yuanzhi OR Yuan Zhi OR Polygala tenuifolia OR Polygala sibirica OR Polygala senega OR Seneca snakeroot OR Milkwort OR Polygala root OR Polygalae Radix OR Radix Polygalae) AND (Acorus OR Shichangpu OR Shi Chang Pu OR Acori Tatarinowii Rhizoma OR Acorus gramineus OR Acorus tatarinowii Schott OR Acorus tatarinowii OR Acorus calamus).” A manual search for all retrieved potentially relevant articles and references lists was carried out to find additional available articles. The complete search strategy used is described in Supplemental Material S2.

### Study selection

Two authors (JZT and MQW) independently assessed eligibility for inclusion by screening titles, abstracts, and full texts according to specified inclusion and exclusion criteria. Any disagreements were discussed to be resolved. In cases where consensus could not be reached, a third author (JS) was consulted. All reasons for exclusion were recorded.

### Data extraction

Two authors (JNN and TL) independently extracted information using a pre-specified form of information extraction. Information to be derived from the studies obtained from the screening includes the lead authors of the paper, the year of publication, participants, age, sex, ethnicity, AD diagnostic criteria, inclusion criteria, specific interventions, treatment duration, outcome indicators, and occurrence of adverse events.

### Primary outcome (cognitive function and daily living skills)

The primary outcomes assessed in this investigation were mini-mental state examination (MMSE), AD assessment scale-cognitive subscale (ADAS-cog), and activities of daily living (ADL).

### Secondary outcome (treatment-associated efficacy and adverse event rates)

The secondary outcomes assessed in this investigation were clinical treatment efficiency, TCM symptom score, and the incidence of adverse events.

### Statistical analysis

#### Assessment of the risk of bias

Two authors (JNN and TL) independently assessed the risk of bias in eligible studies. The Cochrane risk of bias tool was used to assess randomization, allocation concealment, blinding of the intervention, completeness of outcome information, selective reporting, and other sources of bias, such as missed visits or dropout bias. Each item was categorized into three levels, “high risk,” “low risk,” and “unknown risk.” Items that were unclear in the study were further checked by contacting the corresponding author. Again, any disagreements were discussed with a third author (JS). When at least 10 studies were included in the quantitative analysis synthesis, funnel plots were constructed to assess publication bias among studies.

#### Assessment of the quality of the evidence

We chose to use Grading of Recommendations, Assessment, Development, and Evaluations (GRADE) as a criterion for assessing the overall quality of evidence for the outcome indicators of the included trials, which was synthesized in terms of five dimensions: risk of bias, imprecision, inconsistency, indirectness, and publication bias (Schünemann et al., 2019). It was conducted independently by two authors (JZT and YCZ), with disagreements being resolved by a third author (JS).

### Dealing with missing data

We contacted the researchers or authors to obtain unavailable original outcome data when needed. When we did not receive a response, we used only the available data for analysis.

### Measures of treatment effects

All meta-analyses were conducted statistically using RevMan 5.4 and Stata (version 16.0) software. We performed statistical analysis by calculating the MD and the corresponding 95% CI for continuous variable data as effect sizes and risk ratios for dichotomous variable outcomes.

### Assessment of heterogeneity

For statistical heterogeneity, we used the *I* ^*2*^ for testing and assessment. We decided to use a random-effects model for the analysis; or else, when statistical heterogeneity was not significant (*p* > 0.05, *I* ^*2*^ ≤ 50%), we chose to use a fixed-effects model for the analysis. For continuous outcomes and dichotomous outcomes, we performed meta-analysis using the Mantel–Haenszel method and inverse variance method, respectively.

### Subgroup analysis and exploration of heterogeneity

Possible sources of significant heterogeneity were assessed by subgroup analysis and further explored for factors with the greatest influence on high heterogeneity. We performed subgroup analyses of treatment measures and the duration of intervention. If sufficient data were available, the subgroup analysis would be carried out to explore any other effect that might explain any heterogeneity.

### Publication bias

The presence of publication bias was assessed by the observation of the symmetry of the funnel plots, followed by Egger’s test for further statistics and validation (Sterne et al., 2011). Publication bias was assessed using a visual analysis of the funnel plots and Egger’s test when there were at least seven RCTs included in a meta-analysis.

### Sensitivity analysis

Sensitivity analysis was used to examine if significant differences existed in individual studies or outliers that would markedly affect the robustness of overall study outcomes. Statistically significant differences were defined by a *p*-value of < 0.05, which applied to all test results in this study.

## Results

### Results of the search

**FIGURE 1:**
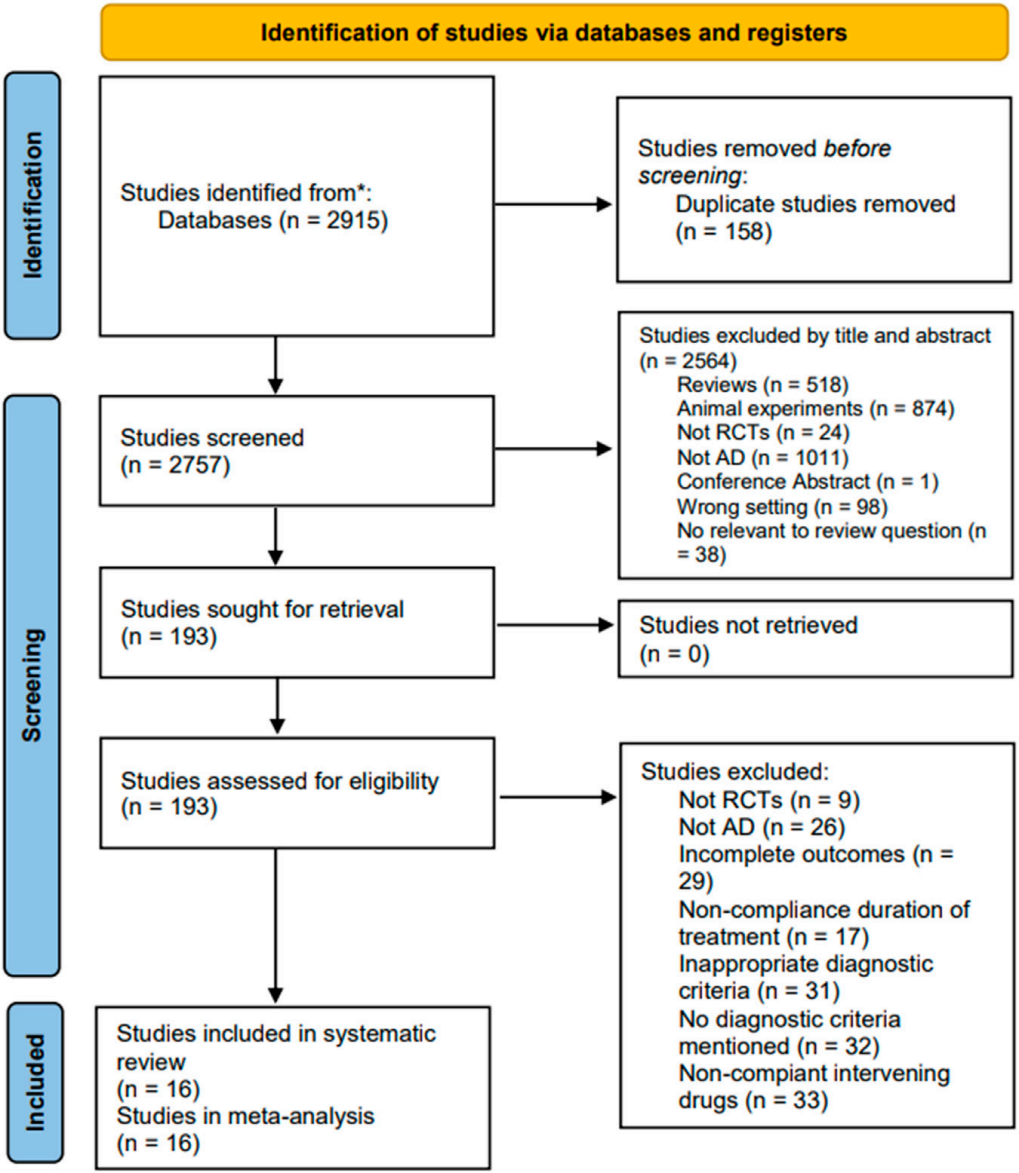
PRISMA flow diagram of research retrieval and the screening process.

Following an initial comprehensive search, 2195 eligible articles were identified. Subsequently, 158 duplicates were removed, and 2564 articles were excluded after a meticulous review of titles and abstracts. Further detailed reading of the full text content of 193 articles led to the final inclusion of 16 articles. The study retrieval process is illustrated in Figure 1.

A total of 16 RCTs were eventually included in this meta-analysis, which involved 1103 AD participants (Table 1). Among all included studies, six trials compared the combination of PT and AT alone with drugs; five of the trials used donepezil, and one used piracetam for the control intervention drug. Ten trials compared the combination of PT and AT plus drugs with drugs, with nine trials using donepezil and one trial using memantine in combination with oxiracetam as the control intervention drug. Eight studies reported the inclusion of subjects with mild-to-moderate AD, and the remaining studies failed to report. Nine studies had a 3-month treatment duration, one study had a 4-month treatment duration, and six studies had a 6-month treatment duration (Table 2). Specific basic information on the herbal prescription ingredients reported in all included trials is provided in Supplemental Material S1.

**TABLE 1:** Characteristics and data used for the studies included in this meta-analysis.

Study ID	Country	Setting	Sample size (T/C)	Sex (M/F)		Mean age (years) (T/C)	Course of disease (T/C)	Experimental group	Control group
				T	C				
YANG et al. (2022)	China	NR	100(50/50)	28/22	26/24	57.10 ± 8.71/59.86 ± 7.81	NR	*Jieyu Yizhi Tang*, bid	Donepezil pill, 5mg, po, qd
WANG et al. (2022)	China	Outpatient	70(35/35)	19/16	17/18	68.91 ± 6.77/69.66 ± 6.66	6.66 ± 3.76/6.49 ± 3.16	*Yizhi Qixin Tang*, bid + control	Donepezil pill, 10mg, po, qd
GUAN (2022)	China	Outpatient + inpatient	65(33/32)	18/15	13/19	72.6 ± 3.16/71.0 ± 3.76	24.21 ± 10.16/20.50 ± 10.10 m	*Modified Kaixin San* + control	Donepezil pill, 5mg, po, qd
WANG (2021)	China	NR	80(40/40)	13/27	15/25	74.78 ± 5.29/75.06 ± 5.15	2.05 ± 0.76/2.10 ± 0.69	*Bushen Huoxue Huatan Tang* + control	Memantine tablet, 5mg, po, qd-bid + oxiracetam, 0.8g, po, tid
YANG et al. (2020)	China	Outpatient + inpatient	84(42/42)	23/19	25/17	71.83 ± 7.98/70.75 ± 7.23	5.44 ± 2.07/5.31 ± 1.14	*Huanshaodan*, bid	Donepezil pill, 5mg, po, qd
GU (2019)	China	Inpatient	60(30/30)	15/15	16/14	80.87 ± 6.60/83.50 ± 7.89	4.54 ± 2.18/4.87 ± 2.35	*Jiawei Diankuang Mengxing decoction* + control	Donepezil pill, 5–10mg, po, qd
Kelaimujiang (2019)	China	Inpatient	66(44/22)	22/22	12/10	76.18 ± 6.3/78.73 ± 5.56	4.50 ± 1.40/4.43 ± 1.43	*Yizhi Chidai recipe* + control	Donepezil pill, 5mg, po, qd
ZHANG et al. (2018)	China	Outpatient + inpatient	60(30/30)	10/20	11/19	66.4 ± 3.3/65.2 ± 4.1	NR	Dihuang Yinzi decoction	Donepezil pill, 5mg, po, qd
YANG et al. (2018)	China	NR	59(31/28)	17/14	15/13	72.66 ± 15.98/73.12 ± 17.65	6.8 ± 2.2/6.0 ± 2.0	Tiaoxin prescription	Donepezil pill, 5mg, po, qd
LIN and CHEN (2018)	China	Outpatient	104(52/52)	31/22	28/25	68.43 ± 7.76/69.59 ± 8.24	NR	*Kaixin San* + control	Donepezil pill, 5mg, po, qd
ZHENG (2017)	China	Inpatient	62(32/30)	19/13	14/16	72.03 ± 7.14/72.20 ± 6.45	NR	*XingZhiSan* + control	Donepezil pill, 5mg, po, qd
LIN (2017)	China	Outpatient + inpatient	60(30/30)	12/18	15/15	62.33 ± 4.74/64.57 ± 5.51	31.94 ± 10.74/32.50 ± 10.82 m	*Huatan Tongqiao decoction* + dontrol	Donepezil pill, 5mg, po, qd
LI (2017)	China	Outpatient + inpatient	63(31/32)	12/19	13/19	74.82 ± 7.96/75.06 ± 7.83	14.17 ± 5.07/15.13 ± 5.73 m	Modified Shuyu pill	Donepezil pill, 5mg, po, qd
PENG (2014)	China	Outpatient + inpatient	30(15/15)	7/8/	6/9	69.87 ± 6.09/69.67 ± 5.92	NR	*Kaixin Jiannao granule* + control	Donepezil pill, 5mg, po, qd
LIANG et al. (2010)	China	Outpatient + inpatient	100(50/50)	21/29	19/31	72.60 ± 7.20/71.7 ± 6.90	6.14 ± 2.41/5.73 ± 2.50	*Bushenyizhi granule* + control	Donepezil pill, 5–10mg, po, qd
CHEN and YUAN (2008)	China	Outpatient	40(20/20)	6/14	5/15	70.0 ± 4.1/70.9 ± 4.7	NR	*Huo Nao Fang*	Piracetam 0.8g, po, bid

T: experimental group; C: control group; M: male; F: female; m: month; NR: not reported.

**TABLE 2:** Characteristics and data used for the studies included in this meta-analysis.

Study ID	Species and amount	Dosage form	Control intervention	Treatment Duration	TCM syndrome differentiation	Outcome measures
Xiong et al. (2022a)	PT 12g, AT 15g	Water decoction	Donepezil pill, 5 mg, po, qd	12 w	Syndrome of liver depression and spleen deficiency	①②④⑥
Jiao et al. (2022)	PT 10g, AT 10g	Machine decoction	Donepezil pill, 10 mg, po, qd	12 w	Kidney essence deficiency and spleen kidney deficiency syndrome	①②③⑤
Bo et al. (2022)	PT 9g, AT 15g	Water decoction	Donepezil pill, 5 mg, po, qd	24 w	Spleen deficiency and sputum resistance type	①②③④
Yi et al. (2018)	PT 15g, AT 15g	Water decoction	Memantine tablet, 5 mg, po, qd-bid + oxiracetam, 0.8g, po, tid	12 w	Kidney deficiency and phlegm stasis type	①④
Wang et al. (2012)	NR	Chinese patent drug	Donepezil pill, 5 mg, po, qd	24 w	Spleen and kidney deficiency type	①②③⑦⑧⑨
Schünemann et al. (2019)	PT 10g, AT 10g	Machine decoction	Donepezil pill, 5–10 mg, po, qd	3 m	Phlegm–stasis syndrome	①②④
Sterne et al. (2011)	PT 10g, AT 10g	Water decoction	Donepezil pill, 5 mg, po, qd	3 m	Kidney essence deficiency type	①④
YANG et al. (2022)	PT 15g, AT 15g	Granules	Donepezil pill, 5 mg, po, qd	3 m	Syndrome of kidney yin yang deficiency combined with phlegm turbidity and obstruction of the orifice	①②③
WANG et al. (2022)	NR	Granules	Donepezil pill, 5 mg, po, qd	3 m	Heart qi deficiency syndrome	①
GUAN (2022)	PT 5g, AT 2.5g	Powder	Donepezil pill, 5 mg, po, qd	24 w	Syndrome of insufficient medullary sea and syndrome of turbid phlegm obstructing the orifices	①
WANG (2021)	PT 10g, AT 20g	Machine decoction	Donepezil pill, 5 mg, po, qd	12 w	Kidney deficiency and phlegm stasis type	①②④
YANG et al. (2020)	PT 6g, AT 6g	Machine decoction	Donepezil pill, 5 mg, po, qd	6 m	Syndrome of phlegm obstructing the orifices	①②③④
GU (2019)	NR	Concentrated decoction	Donepezil pill, 5 mg, po, qd	12 w	Kidney deficiency and marrow depletion syndrome	①②③⑧
Kelaimujiang (2019)	PT 10g, AT 5g	Granules	Donepezil pill, 5 mg, po, qd	4 m	Spleen and kidney deficiency, sputum turbid blocking aperture syndrome	①②④
ZHANG et al. (2018)	PT 6g, AT 6g	Granules	Donepezil pill, 5–10 mg, po, qd	24 w	Kidney deficiency and blood stasis syndrome	①②③④
YANG et al. (2018)	NR	Machine decoction	Piracetam 0.8 g, po, bid	6 m	Kidney deficiency and marrow depletion syndrome	①④

① MMSE; ② ADL; ③ ADAS-cog; ④ TCM symptom score; ⑤ SOD; ⑥ Hcy; ⑦ Mo CA; ⑧SDSD; ⑨ CDR; PT: *Polygala tenuifolia*; AT: *Acorus tatarinowii*; NR: not reported; po: per os; qd: once a day; bid: twice a day; mg: milligram; g: gram; m: month; w: week; TCM: traditional Chinese medicine.

### Methodological quality assessment

**FIGURE 2:**
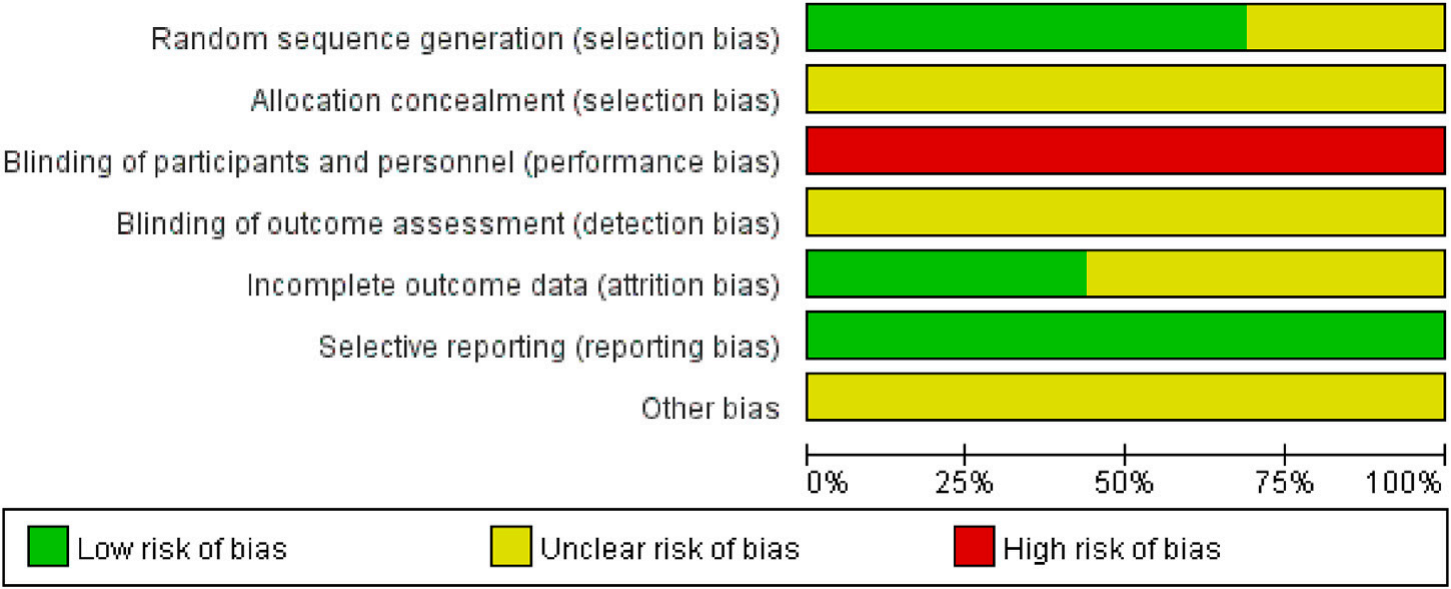
Risk of bias graph.

A total of 16 studies were included for the risk of bias assessment. In the detailed assessment, 11 studies were assessed to be at low risk during randomization, and the remaining five studies were assessed to be at unknown risk because they were described as “randomized” without specifying the exact method of randomization. Regarding blinding of participants and personnel, all studies were assessed as high risk. One study suggested that the blinding method should be single-blind, and the allocation of the remaining studies was unclear in terms of concealment of allocation, the blinding method, and trial implementation. All studies were considered to have a low risk of bias in the domain of selective reporting. As for the incomplete outcome data, nine studies were assessed as having an unknown risk because they were not explicitly reported, and five studies were low risk. All studies did not explicitly mention detection bias and other bias and were assessed as having an unknown risk. The results of the risk of bias evaluation are shown in Figure 2 and Supplemental Material S3.

### Meta-analysis

#### Primary outcome (cognitive function)

##### MMSE

Sixteen studies were analyzed using MMSE as an evaluation metric, totaling 1103 subjects. The effects of the combination of PT and AT on AD were not significantly different in terms of MMSE when compared to conventional drug therapy [MD = 0.33, 95%CI: (−0.64, 1.31); *p* = 0.5; *I* ^*2*^ = 42%]. We found a significant reduction in heterogeneity (42% vs. 8%) after excluding Chen’s study, which may be because piracetam was chosen as the intervention drug for the control group in this trial, whereas all the other studies used donepezil at the same dose and dosage. The combination of PT and AT plus control was more effective in improving MMSE compared to control [MD = 2.57, 95%CI: (1.44, 3.69); *p* < 0.00001; *I* ^*2*^ = 86%] (Table 3; Supplemental Material S4, Supplementary Figure S1).

**TABLE 3:** Effects of the combination of PT and AT on AD from 16 randomized controlled trials.

Outcome	No. of studies	No. of participants	I^2^, model (REM/FEM)	Effect size	Quality of evidence (GRADE)
				MD [95% CI]	
*PT and AT* versus control					
MMSE	6	406	42%, REM	0.33 [−0.64, 1.31]	Very low certainty
ADL	3	207	0%, FEM	−0.83 [−1.73, 0.08]	Very low certainty
ADAS-cog	3	207	0%, FEM	−0.66 [−1.69, 0.37]	Very low certainty
TCM symptom score	2	140	0%, REM	−3.08 [−4.51, −1.64]	Low certainty
*PT and AT* plus control versus control					
MMSE	10	697	86%, REM	2.57 [1.44, 3.69]	Very low certainty
ADL	6	377	0%, FEM	−3.19 [−4.29, −2.09]	Moderate certainty
ADAS-cog	4	295	0%, FEM	−2.09 [−3.07, −1.10]	Low certainty
TCM symptom score	7	461	80%, REM	−4.32 [−5.89, −2.75]	Very low certainty

PT: *Polygala tenuifolia;* AT: *Acorus tatarinowii;* MD: mean difference; CI: confidence interval; No: number; REM: random-effects model; FEM: fixed-effects model; MMSE: mini-mental state examination; ADL: activities of daily living; ADAS-cog: AD assessment scale-cognitive subscale; TCM: traditional Chinese medicine.

The results of the subgroup analyses indicated that treatment duration was not a source of significant heterogeneity. Compared with the control group, there was no significant difference in the effect of the combination of PT and AT on MMSE at a treatment duration of 3 months (MD = −0.28, 95%CI: [−0.98, 0.43]; *p* = 0.44; *I* ^*2*^ = 0%), whereas the combination of PT and AT plus drugs was superior to the use of drugs alone (MD = 2.55, 95%CI: [0.96, 4.14]; *p* = 0.002; *I* ^*2*^ = 92%). At a treatment duration of 6 months, the combination of PT and AT still appeared to show less effect on MMSE than the control group (MD = 1.72, 95%CI: [0.01, 3.42]; *p* = 0.05; *I* ^*2*^ = 29%), while the combination of PT and AT plus drugs remained superior to the use of drugs alone (MD = 2.27, 95%CI: [0.81, 3.74]; *p* = 0.002; *I* ^*2*^ = 49%) (Table 4; Supplemental Material S4, Supplementary Figures S5, 8).

**TABLE 4:** Results of the subgroup analysis.

Outcome	Subgroup	No. of studies	No. of participants	I^2^, model (REM/FEM)	Effect size	Quality of evidence (GRADE)
	Treatment duration(m)				MD [95% CI]	
*PT and AT* versus control						
MMSE	3	4	282	0%, FEM	−0.28 [−0.98, 0.43]	Very low certainty.
	6	2	124	29%, FEM	1.72 [0.01, 3.42]	Low certainty.
ADL	3	2	123	0%, FEM	−0.56 [−1.59, 0.46]	Very low certainty.
	6	1	84	NA	−1.76 [−3.69, 0.17]	Very low certainty.
ADAS-cog	3	2	123	0%, FEM	−0.80 [−2.17, 0.58]	Very low certainty.
	6	1	84	0%, FEM	−0.49 [−2.05, 1.07]	Very low certainty.
*PT and AT* plus control versus control						
MMSE	3	5	338	92%, REM	2.55 [0.96, 4.14]	Very low certainty.
	4	1	30	NA	3.33 [0.19, 6.47]	Very low certainty.
	6	4	329	49%, REM	2.27 [0.81, 3.74]	Low certainty.
ADL	3	2	122	0%, FEM	−2.38 [−5.34, 0.59]	Very low certainty.
	4	1	30	NA	−6.79 [−12.16, −1.42]	Very low certainty.
	6	3	225	0%, FEM	−3.14 [−4.36, −1.92]	Low certainty.
ADAS-cog	3	1	70	NA	−2.40 [−4.38, −0.42]	Very low certainty.
	6	3	225	0%, FEM	−1.98 [−3.12, −0.84]	Low certainty.
TCM symptom score	3	3	206	71%, REM	−4.38 [-6.16, −2.60]	Very low certainty.
	4	1	30	NA	−8.00 [−15.46, −0.54]	Very low certainty.
	6	3	225	91%, REM	−4.03 [−7.36, −0.69]	Very low certainty.

PT: *Polygala tenuifolia;* AT: *Acorus tatarinowii;* MD: mean difference; CI: confidence interval; No: number; REM: random-effects model; FEM: fixed-effects model; MMSE: mini-mental state examination; ADL: activities of daily living; ADAS-cog: AD assessment scale-cognitive subscale; TCM: traditional Chinese medicine.

##### ADAS-cog

A meta-analysis of seven randomized controlled trials including 502 patients showed no statistically significant differences in the effects of the combination of PT and AT on ADAS-cog compared to conventional drug in the control group (MD = −0.66, 95%CI: [−1.69, −0.37]; *p* = 0.21; *I* ^*2*^ = 0%). The combination of PT and AT plus conventional drugs was more effective in improving ADAS-cog compared to Western drugs (MD = −2.09, 95%CI: [−3.07, −1.10]; *p* < 0.0001; *I* ^*2*^ = 0%) (Table 3; Supplemental Material S4, Supplementary Figure S3).

Subgroup analyses of treatment duration showed that at 3 months of treatment, the combination of PT and AT was not as effective as conventional drug therapy for ADAS-cog [MD = −0.80, 95%CI: (−2.17, 0.58); *p* = 0.26; *I* ^*2*^ = 0%]. At 6 months of treatment, the combination of PT and AT plus drugs was superior for ADAS-cog [MD = −1.98, 95%CI: (−3.12, −0.84); *p* = 0.0007; *I* ^*2*^ = 0%] (Table 4; Supplemental Material S4, Supplementary Figures S7, 10).

#### Primary outcome (daily living skills)

##### ADL

Eight studies, involving 584 participants, used ADL scores as an evaluation metric. The results of meta-analysis using a fixed-effects model showed no statistically significant difference in the effect of the combination of PT and AT on ADL compared with conventional drug therapy [MD = −0.83, 95%CI: (−1.73, 0.08); *p* = 0.07; *I* ^*2*^ = 0%]. The combination of PT and AT plus drugs showed significant beneficial effects on ADL scores compared to drugs [MD = −3.19, 95%CI: (−4.29, −2.09); *p* < 0.00001; *I* ^*2*^ = 0%] (Table 3; Supplemental Material S4, Supplementary Figure S2).

The results of the subgroup analyses showed no significant difference in the therapeutic effect of the combination of PT and AT on ADL in AD patients compared with drugs at a treatment duration of 3 months [MD = −0.56, 95%CI: (−1.59, 0.46); *p* = 0.28; *I* ^*2*^ = 0%]. At a treatment duration of 3 months, there was no significant difference in the benefits of the combination of PT and AT plus conventional drug therapy on ADL in AD patients compared with conventional drug therapy [MD = −2.38, 95%CI: (−5.34, 0.59); *p* = 0.12; *I* ^*2*^ = 0%]. At a treatment duration of 6 months, the combination of PT and AT plus control treatment had a significant potentiating effect on ADLs in AD patients [MD = −3.14, 95%CI: (−4.36, −1.92); *p* < 0.00001; *I* ^*2*^ = 0%] (Table 4; Supplemental Material S4, Supplementary Figures S6, 9).

#### Secondary outcome (treatment-associated efficacy)

##### TCM symptom score

Nine studies involving 601 subjects referred to the TCM symptom score, and the results of the random-effects model showed that the combination of PT and AT had a superior effect on the TCM symptom score [MD = −3.08, 95%CI: (−4.51, −1.64); *p* < 0.0001; *I* ^*2*^ = 0%] compared to the control group treated with conventional drugs, while the combination of PT and AT plus conventional drugs also showed more beneficial effects [MD = −4.32, 95%CI: (−5.89, −2.75); *p* < 0.00001; *I* ^*2*^ = 80%] (Table 3; Supplemental Material S4, Supplementary Figure S4).

Subgroup analyses of treatment duration showed that the combination of PT and AT plus conventional drugs in the control group improved the TCM symptom score significantly better than the control group, both at 3 months [MD = −4.38, 95%CI: (−6.16, −2.60); *p* < 0.00001; *I* ^*2*^ = 71%] and 6 months [MD = −4.03, 95%CI: (−7.36, −0.69); *p* = 0.02; *I* ^*2*^ = 91%] of treatment (Table 4, Supplemental Material S4, Supplementary Figure S11.

##### Clinical efficacy

Only one study characterized the clinical effectiveness of the combination of PT and AT versus conventional drugs; therefore, meta-analysis could not be performed. Five studies reported the clinical effectiveness of the combination of PT and AT plus conventional drugs, involving a total of 401 subjects. The results showed that the combination of PT and AT plus conventional drugs had a significantly more favorable benefit in terms of clinical effectiveness [RR = 1.27, 95%CI: (1.12, 1.44); *p* = 0.0002; *I* ^*2*^ = 0%] (Supplemental Material S4, Supplementary Figure S14).

#### Secondary outcome (adverse event rates)

##### Safety of the combination of PT and AT

Eight randomized controlled trials containing 604 subjects reported adverse events. Symptoms reported for non-serious adverse events included dizziness and nausea, and symptoms resolved on their own without intervention. No trials reported serious adverse events. The combination of PT and AT showed lower rates of adverse events compared to conventional drug therapy (8.1% vs. 25.8%), while the difference between the combination of PT and AT plus conventional drug therapy was not significant compared to conventional drug therapy (8.3% vs. 13%). Fixed-effects model analysis showed that the adverse event rates for the combination of PT and AT were significantly lower than those for conventional drugs therapy [RR = −0.33, 95%CI: (0.19, 0.60); *p* = 0.0003; *I* ^*2*^ = 0%] (Supplemental Material S4, Supplementary Figure S12). Adverse events were not increased with the combination of PT and AT plus conventional drugs compared to conventional drugs [RR = 0.65, 95%CI: (0.35, 1.19); *p* = 0.16; *I* ^*2*^ = 0%] (Supplemental Material S4, Supplementary Figure S13).

##### Results of sensitivity analysis and publication bias analysis

**FIGURE 3:**
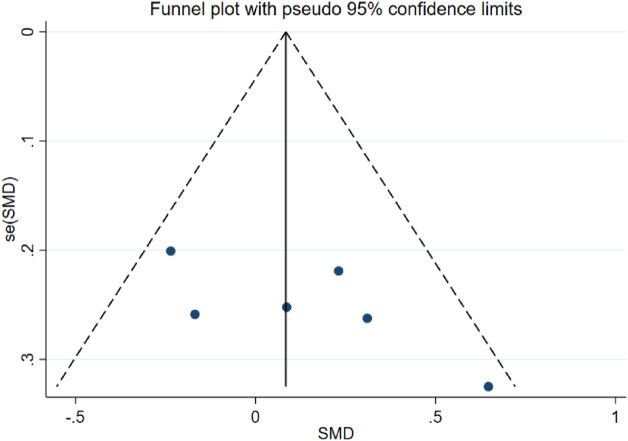
Forest plot of MMSE when compared with the combination of PT and AT with control.

**FIGURE 4:**
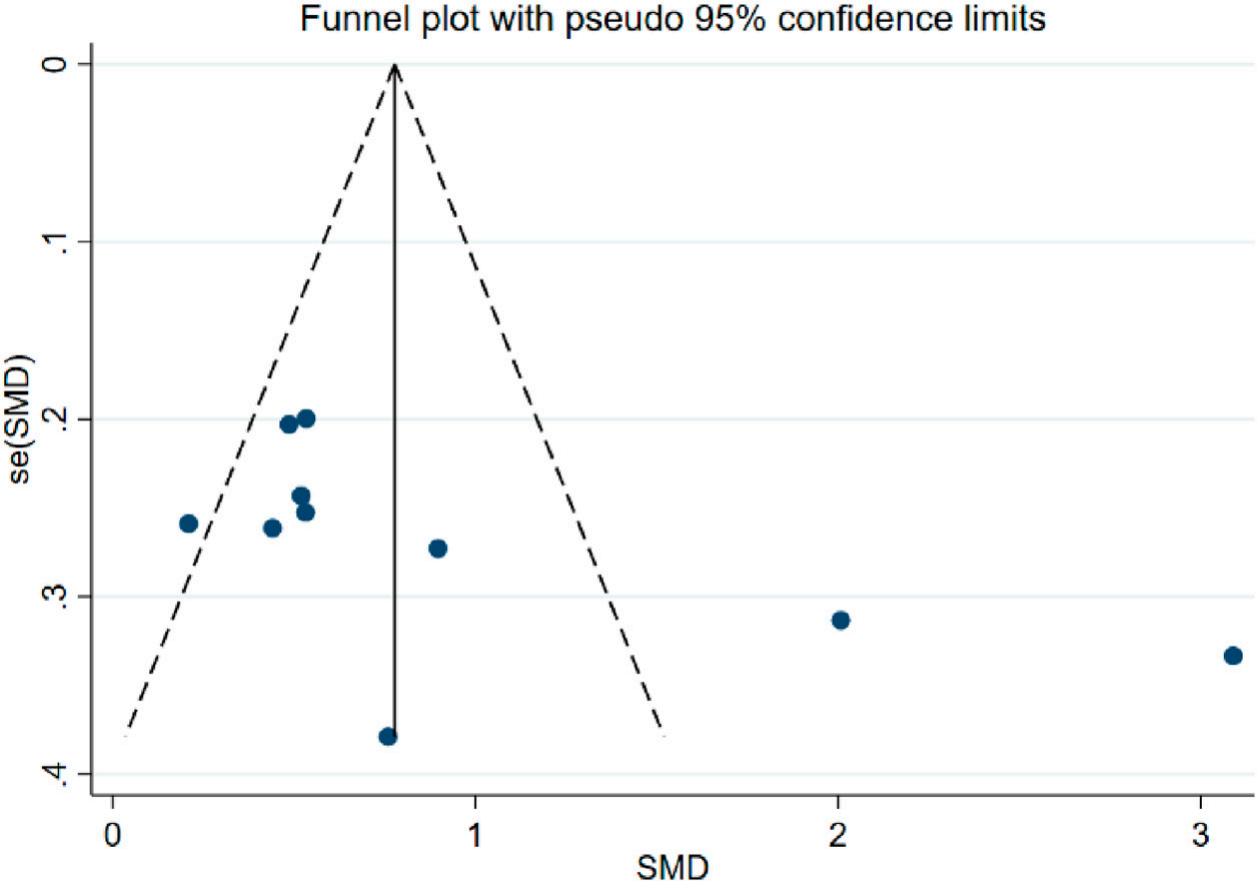
Forest plot of MMSE when compared with the combination of PT and AT plus control with control.

Sensitivity analyses showed that the quality of the included trials did not have an impact on the stability of the results, which remained stable and reliable (Supplemental Material S5, Supplementary Tables S3, 5). Due to the available data, we only assessed the publication bias of the combination of PT and AT on MMSE in AD patients (Zhang et al., 2022). Analysis results of the publication bias of the combination of PT and AT on MMSE by Egger’s test (*p* = 0.119) suggested no publication bias present, and the results of funnel plot assay were consistent with this (Figure 3, Supplemental Material S5; Supplementary Table S4). Visual inspection of the funnel plots showed some asymmetry (Figure 4), and when we assessed the combination of PT and AT plus control on MMSE with Egger’s test (*p* = 0.066), no publication bias was observed (Supplemental Material S5, Supplementary Table S6).

##### Certainty of evidence quality

**FIGURE 5:**
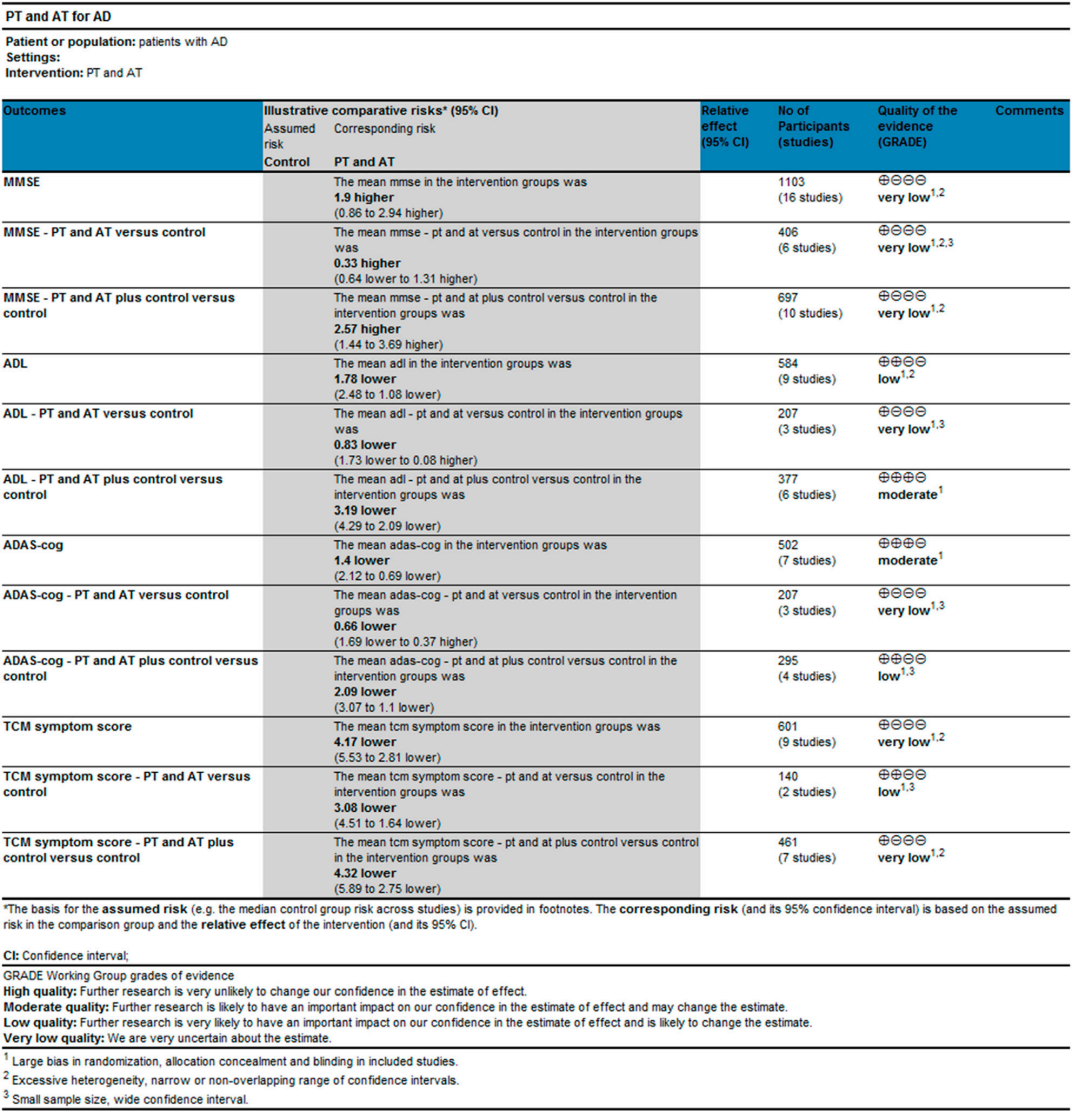
Results of evidence quality evaluation of GRADE for the combination of PT and AT discussion.

Very low-quality evidence suggested that the effects of the combination of PT and AT alone on MMSE, ADLs, and ADAS-cog in patients with AD are comparable to those of conventional drug therapy. Low-quality evidence suggested that the combination of PT and AT has a better effect on TCM symptom scores in AD patients than conventional drugs. Very low-quality evidence showed that the effects of the combination of PT and AT plus conventional drugs in MMSE and TCM symptom scores were comparable to conventional drug treatments. Evidence of low to moderate quality demonstrates that the combination of PT and AT plus conventional drugs is superior to conventional drugs on MMSE, ADL, and ADAS-cog in AD patients (Figure 5). Results of subgroup analyses based on the treatment duration of AD revealed that the quality of evidence for the outcomes of MMSE, ADL, ADAS-cog, and TCM was low or very low. Full details of the evidence summary are in Supplemental Material S6.

## Discussion

### Comparison with other studies or reviews

The pathomechanism of AD is complex, and numerous studies have been conducted but still have not led to drugs and targets that can effectively delay the progression of the disease (Ferrari and Sorbi, 2021; Passeri et al., 2022b). TCM formulas with PT as the principal drug have been repeatedly shown to improve cognitive symptoms in AD by consistently regulating phosphorylated tau (Qu et al., 2021; Li et al., 2023). AT has also been implicated in the therapeutic potential of AD by modulating the BDNF/ERK/CREB signaling pathway and the synapse-associated protein PSD-95 (Ning et al., 2021). The results of modern network pharmacology and experimental studies show that the combination of PT and AT has multi-component characteristics and advantages and can exert inhibitory effects on AD through multiple pathways, such as down-regulating the expression levels of TLR4 and NF-κB and related inflammatory factor targets (e.g., TNF-α, IL-1β, and IL-6) (Su et al., 2022; Wu et al., 2022; Cao et al., 2023).

TCM plays an important role in the clinical treatment of AD due to its unique advantages in combined medication (Tan et al., 2022; Xu et al., 2023). The combination of PT and AT is high-frequency drug pairs in TCM formulas for the treatment of AD. *Polygala tenuifolia* has the effects of tranquilizing the mind and promoting intelligence, coordinating the heart and the kidney, dispelling phlegm, and opening the orifices. *Acorus tatarinowii* is ranked first among the herbaceous medicines in “Shen Nong’s herbal classic,” with the effects of inducing resuscitation, calming the heart tranquilizing the mind, and removing dampness to restore normal functioning of the stomach. We found that out of the 16 RCTs included in this study, 11 were related to “kidney deficiency” in TCM syndrome differentiation, 5 were related to “spleen deficiency,” 8 were related to “phlegm turbidity,” and 4 were related to “blood stasis.” Therefore, we believe that the treatment of AD with the combination of PT and AT conforms to the evolution of TCM syndromes during the progression of AD disease, demonstrating a certain syndrome correlation effect.

### Summary of the main findings

This study evaluated and analyzed the effects of the combination of PT and AT on AD and included 16 RCTs involving 1103 participants. In this review, six trials compared the combination of PT and AT with conventional drugs (five donepezil and one piracetam). Ten RCTs compared the combination of PT and AT plus conventional drugs with conventional drugs (nine donepezil and one memantine + oxiracetam). Meanwhile, AD patients included in this study were mainly mild-to-moderate AD. For the clinical treatment of mild-to-moderate AD, donepezil is still the conventional drug mainly used. The analysis results indicated that the use of the combination of PT and AT alone did not have a significant effect on MMSE, ADL, and ADAS-cog in AD patients. Although the combination of PT and AT alone has shown a trend toward better outcomes with longer interventions, their clinical relevance remains limited. However, the combination of PT and AT plus conventional drugs showed more beneficial effects on MMSE, ADL, and ADAS-cog in patients with AD than drugs alone. In addition, we noted significant heterogeneity in the effects of both the combination of PT and AT and the combination of PT and AT plus conventional drugs on MMSE in patients with AD. By subgroup analysis of treatment duration, we were still unable to determine the source of heterogeneity. Based on the availability of included studies and the adequacy of data, we are currently unable to analyze to determine whether the factors affecting heterogeneity are related to other factors such as the dosage form, dosage, and subjectivity of TCM syndrome differentiation. In terms of safety reports, the combination of PT and AT had fewer adverse events than conventional drugs, while the combination of PT and AT plus conventional drugs was comparable to conventional drugs. In addition, the results of the subgroup analyses suggested that the effects of the combination of PT and AT plus conventional drugs on MMSE, ADL, and ADAS-cog were significant at 6 months of treatment duration, but given the clinical peculiarities of the AD disease course, increasing or adjusting the dosage over time and evolution is necessary, which may also increase the potential for secondary adverse reactions. Moreover, “syndrome” is of great importance in the process of TCM treatment, and it constantly changes with the passage of time and the evolution of the disease process (Li et al., 2021; Lu et al., 2021). Therefore, further prospective randomized controlled trials with large samples and follow-up are needed to assess the long-term efficacy of TCM treatments to avoid the limitation that conventional drug interventions are only effective in the short term.

### Quality of the evidence

We judged the overall quality of evidence for global cognition and functioning to be very low when the combination of PT and AT plus conventional drugs was compared with conventional drugs. The overall quality of evidence for global cognition and functioning was very low to moderate when the combination of PT and AT plus conventional drugs was compared with conventional drugs.

### Implications for practice and future research

In the results of the meta-analyses evaluating the two treatment regimens separately, the direction of the effect sizes associated with the improvement of global cognition and functioning was consistent. In terms of the improvement in global cognition and functioning from the analyses of the included studies, we obtained positive results. The combination of PT and AT can be used as alternative therapies to conventional drugs for the treatment of AD, and the efficacy of the combination of PT and AT plus control is superior. However, the low and very low quality of available evidence limits confidence in the findings.

In our included studies, the maximum treatment duration was 6 months. The efficacy of both the combination of PT and AT and the combination of PT and AT plus control was better at 6 months than at 3 months of treatment duration. Our findings suggest that the combination of PT and AT demonstrated comparable effects to conventional drugs in terms of improvement of AD, with fewer adverse events, and showed a strong trend toward positive effects and marginal significance at 6 months of continuous treatment. With the longer duration of AD, a 6-month treatment duration does not appear to be the optimal period for the combination of PT and AT to be used to cure the disease; however, it still needs to be supported by more evidence, and the results need to be interpreted with caution. In the future, prospective trials may be needed to examine when a ceiling effect occurs in treatment interventions involving the combination of PT and AT.

We also tried to explore whether the therapeutic effect of the combination of PT and AT on AD is related to the amount or dosage form of the herbs, but the data from the existing studies were not sufficient to support us in finding a suitable methodology to analyze it. The bioavailability of the combination of PT and AT alone or in combination with conventional drugs may require more studies to conclude.

If the composition of TCM prescriptions in each study is strictly considered, it may be impossible to quantitatively summarize data from almost all TCM clinical studies. The present study focused on a meta-analysis of the macroscopic assessment of AD with the combination of PT and AT combined with conventional drugs compared with the combination of PT and AT alone. The resulting heterogeneity has been a methodological challenge for meta-analysis in TCM clinical studies (Dong et al., 2017).

In addition, it has been reported in some studies that TCM may have potential toxicity in clinical applications (Teo et al., 2016; Pan et al., 2020). Studies have shown that no toxic reactions were observed with PT and AT in relevant cell and animal experiments (Chen and Jia, 2020; Xiong et al., 2022b; Chen et al., 2022). Our findings suggested that the combination of PT and AT has fewer adverse effects in AD treatment and is safer for clinical application. It is also worth mentioning that the combination of PT and AT plus control showed a trend of fewer adverse reactions compared to when conventional drugs were applied. With the prolongation of the disease duration and treatment cycle of AD, the increase in the dosage of conventional drugs is inevitable (Cummings et al., 2019), and the combination of PT and AT may be beneficial in reducing the side effects of conventional drugs. More rigorous clinical trials are needed to explore the optimal dosage and timing of the combination of PT and AT use for benefit during different stages of AD.

### Strengths

To our knowledge, this is the first systematic review and meta-analysis to assess the effect of the combination of PT and AT on AD. We followed strictly the Cochrane methodology and conducted systematic and comprehensive searches in different databases. We compared different interventions and treatment durations separately based on the specificity of the AD disease itself and TCM treatment and focused on TCM syndrome differentiation during AD treatment. In addition, we carefully screened the data for each indicator in the combined analyses of the included studies, and we excluded three studies that used different ADL scoring criteria that resulted in opposite clinically significant outcomes to ensure homogeneity of findings.

### Limitations

This study still had several limitations. Due to the multi-component and multi-target nature of TCM treatments, we were unable to clarify the dose relationship between the intervention doses of the combination of PT and AT and changes in outcomes. The combination of PT and AT may not always be used as a principal drug in TCM formulas, and we are unable to analyze whether other active ingredients that improve outcomes in AD patients are also included in the formulas so that TCM treatments may show better improvement. The limitations of risks of bias that existed in the original study may have reduced the quality of the evidence, but we aimed to explore the effects of the combination of PT and AT in patients with AD, and therefore, this study is still of importance. In addition, the treatment duration in this study was a maximum of 6 months, and more RCTs with longer treatment durations are needed in the future to determine how effective the combination of PT and AT is for long-term intervention in AD. Given these publication biases that cannot be completely excluded, we should be cautious in interpreting the results. Sample sizes should be increased and longer treatment durations should be available in future RCTs to improve test efficacy. The design should be further refined in future RCTs, with more rigorous inclusion and exclusion criteria for interventions and reporting of more explicit quality control of prescriptions and chemical analyses studies of drugs.

## Conclusion

Compared to single conventional drugs, the combination of PT and AT may be used as an alternative therapy to improve global cognition and functioning in AD, and the combination of PT and AT as an adjunctive therapy appears to produce a better therapeutic response to AD in terms of efficacy without increasing the risk of adverse events. However, the very low to low quality of available evidence limits confidence in the findings. More prospective studies with tightly controlled conditions are needed to provide confirmatory evidence.

## Data Availability

The original contributions presented in the study are included in the article/Supplementary Material; further inquiries can be directed to the corresponding author.
