# Supplementary material for: Polygala tenuifolia and Acorus tatarinowii in the treatment of Alzheimer’s disease: a systematic review and meta-analysis
Source: Front Pharmacol. 2024 Jan 12;14:1268000. doi: 10.3389/fphar.2023.1268000 (PMC10815298; doi:10.3389/fphar.2023.1268000)
Supplement: Supplementary file 1 [file Table1.docx]

**Table 1 The ingredients of the Chinese herbal prescriptions in the included 16 studies**

| Study ID | TCM syndrome differentiation | TCM prescriptions | Composition of TCM prescriptions | |
| --- | --- | --- | --- | --- |
|  |  |  | Latin name | Chinese name |
| Yang L 2022 | Syndrome of Liver Depression and Spleen Deficiency | *Jieyu Yizhi tang* | *Polygala tenuifolia 12g, Acorus tatarinowii 15g, radix bupleuri 12g, Codonopsis pilosula 30g, Angelica sinensis 30g, Poria cocos 30g, Rhizoma Atractylodis Macrocephalae 30g, Schisandra chinensis 30g, Rhizoma Pinellinae Praeparata 9g, White peony 15g, liquorice 10g* | Yuanzhi 12g, Shichangpu 15g, Chaihu 12g, Dangshen 30g, Danggui 30g, Fuling 30g, Fuchaobaizhu 30g, Wuweizi 30g, Fabanxia 9g, Baishao 15g, Gancao 10g |
| Wang SL 2022 | Kidney essence deficiency and spleen kidney deficiency syndrome | *Yizhi Qixin tang* | *Polygala tenuifolia 10g, Acorus tatarinowii 10g, prepared rehmannia root 30g, tortoise plastron 10g, Cornus officinalis 10g, the fruit of Chinese wolfberry 20g, Atractylodes macrocephala 15g, Poria cocos 15g, Codonopsis pilosula 15g, arisaema with bile 3g, prepared licorice 6g* | Yuanzhi 10g, Shichangpu 10g, Shudihuang 30g, Guiban 10g, Shanyurou 10g, Gouqizi 20g, Baizhu 15g, Fuling 15g, Dangshen 15g, Zhinanxing 3g, Zhigancao 6g |
| Guan XJ 2022 | Spleen Deficiency and Sputum Resistance Type | *Modified Kaixin San* | *Polygala tenuifolia 9g, Acorus tatarinowii 15g, ginseng 12g, Poria cocos 18g, Pinellia ternata 9g, Astragalus membranaceus 30g, Atractylodes macrocephala 10g, Alpiniae Oxyphyllae Fructus 20g, Rhizoma Dioscoreae 30g, prepared licorice 6g* | Yuanzhi 9g, Shichangpu 15g, Renshen 12g, Fuling 18g, Banxia 9g, Huangqi 30g, Baizhu 10g, Yizhiren 20g, Shanyao 30g, Zhigancao 6g |
| Wang P 2021 | Kidney deficiency and phlegm stasis type | *Bushen Huoxue Huatan Tang* | *Polygala tenuifolia 15g, Acorus tatarinowii 15g, prepared rehmannia root 20g, Ligustrum lucidum 20g, the fruit of Chinese wolfberry 20g, Dodder 20g, Cornus officinalis 20g, Salvia miltiorrhiza 15g, Red peony 15g, tuber fleeceflower stem 30g, fossil fragments 30g, pericarpium citri reticulatae 10g, Radix liquiritiae 6g* | Yuanzhi 15g, Shichangpu 15g, Shudihuang 20g, Nvzhenzi 20, Gouqizi 20g, Tusizi 20g, Shanzhuyu 20g, Danshen 15g, Chishao 15g, Yejiaoteng 30g, Longgu 30g, Chenpi 10g, Gancao 6g |
| Yang F 2020 | spleen and kidney deficiency type | *Huanshaodan* | *Polygala tenuifolia, Acorus tatarinowii, prepared rehmannia root, Cornus officinalis, the fruit of Chinese wolfberry, Schisandra chinensis, Cistanche deserticola, Morinda officinalis, fennel, Eucommia ulmoides, radix achyranthis bidentatae, Fructus Broussonetiae, Poria cocos, yam, Jujube* | Yuanzhi, Shichangpu, Shudihuang, Shanzhuyu, Gouqizi, Wuweizi, Roucongrong, Bajitian, Xiaohuixiang, Duzhong, Niuxi, Chushizi, Fuling, Shanyao, Dazao |
| Gu YL 2019 | Phlegm-stasis syndrome | *Jiawei Diankuang Mengxing Decoction* | *Polygala tenuifolia 10g, Acorus tatarinowii 10g, peach kernel 15g, radix bupleuri 9g, cyperus rotundus 6g, akebia 9g, Red peony 9g, Pinellia ternata 6g, pericarpium arecae 9g, Green skin 6g, pericarpium citri reticulatae 9g, Morus alba 9g, Perilla seed 12g, Radix liquiritiae 15g* | Yuanzhi 10g, Shichangpu 10g, Taoren 15g, Chaihu 9g, Xiangfu 6g, Mutong 9g, Chishao 9g, Banxia 6g, Dafupi 9g, Qingpi 6g, Chenpi 9g, Sangbaipi 9g, Zisuzi 12g, Gancao 15g |
| Guzainuer 2019 | kidney essence deficiency type | *Yizhi Chidai recipe* | *Polygala tenuifolia 10g, Acorus tatarinowii 10g, Rehmannia glutinosa 15g, Cornus officinalis 15g, Astragalus membranaceus 15g, radix curcumae 10g, Angelica sinensis 10g, Ligusticum wallichii 10g, Amomum amarum 15g, Deerhorn glue 15g, wine-treated rhubarb 6g* | Yuanzhi 10g, Shichangpu 10g, Dihuang 15g, Shanzhuyu 15g, Huangqi 15g, Yujin 10g, Danggui10g, Chuanxiong 10g, Yizhizi 15g, Lujiaojiao 15g, Jiudahuang 6g |
| Zhang L 2018 | Syndrome of Kidney Yin Yang Deficiency Combined with Phlegm Turbidity and Obstruction of the Orifice | *Dihuang Yinzi Decoction* | *Polygala tenuifolia 15g, Acorus tatarinowii 15g, prepared rehmannia root 15g, Cornus officinalis 15g, Ophiopogon japonicus 15g, Schisandra chinensis 6g, dendrobe 12g, Cistanche deserticola 15g, Morinda officinalis 15g, Cooked Aconite 9g, Cinnamomum cassia 3g, Poria cocos 15g, ginger 6g, Jujube 10g, mint 6g* | Yuanzhi 15g, Shichangpu 15g, Shudihuang 15g, Shanzhuyu 15g, Maidong 15g, Wuweizi 6g, Shihu 12g, Roucongrong 15g, Bajitian 15g, Shufuzi 9g, Rougui 3g, Fuling 15g, Shengjiang 6g, Dazao 10g, Bohe 6g |
| Yang XC 2018 | Heart Qi Deficiency Syndrome | *Tiaoxin Prescription* | *Polygala tenuifolia, Acorus tatarinowii, Astragalus membranaceus, radix bupleuri, prepared licorice* | Yuanzhi, Shichangpu, Huangqi, Chaihu, Zhigancao |
| Ling DX 2018 | Syndrome of insufficient medullary sea and syndrome of turbid phlegm obstructing the orifices | *Kaixin San* | *Polygala tenuifolia 5g, Acorus tatarinowii 2.5g, ginseng 5g, Poria cocos 5g* | Yuanzhi 5g, Shichangpu 2.5g, Renshen 5g, Fuling 5g |
| Zheng R 2017 | Kidney deficiency and phlegm stasis type | *XingZhiSan* | *Polygala tenuifolia 10g, Acorus tatarinowii 20g, ginseng 10g, Morinda officinalis 10g, Salvia miltiorrhiza 30g, Poria cocos 15g* | Yuanzhi 10g, Shichangpu 20g, Renshen 10g, Bajitian 10g, Danshen 30g, Fuling 15g |
| Lin YQ 2017 | Syndrome of phlegm obstructing orifices | *Huatan Tongqiao Decoction* | *Polygala tenuifolia 6g, Acorus tatarinowii 6g, arisaema with bile 6g, Pinellia ternata 5g, earthworm 10g, Astragalus membranaceus 10g, Cistanche deserticola 10g, Poria cocos 10g, pericarpium citri reticulatae 3g, dark plum 1, Radix liquiritiae 3g* | Yuanzhi 6g, Shichangpu 6g, Dannanxing 6g, Zhibanxia 5g, Dilong 10g, Huangqi 10g, Roucongrong 10g, Fuling 10g, Chenpi 3g, Wumei 1, Gancao 3g |
| Li XW 2017 | Kidney Deficiency and Marrow Depletion Syndrome | *Modified Shuyu Pill* | *Polygala tenuifolia, Acorus tatarinowii, Rhizoma Dioscoreae, prepared rehmannia root, Eucommia ulmoides, the fruit of Chinese wolfberry, Poria cocos, et al* | Yuanzhi, Shichangpu, Shanyao, Shudi, Duzhong, Gouqi, Fuling, et al |
| Peng XM 2014 | Spleen and Kidney Deficiency, Sputum Turbid Blocking Aperture Syndrome | *Kaixin Jiannao Granule* | *Polygala tenuifolia 10g, Acorus tatarinowii 5g, Cistanche deserticola 15g, rhodiola root 10g, rhizoma curcumae longae 10g, ginseng 5g, Poria cocos 15g, chlorite schist 2g* | Yuanzhi 10g, Shichangpu 5g, Roucongrong 15g, Hongjingtian 10g, Jianghuang 10g, Renshen 5g, Fuling 15g, Mengshi 2g |
| Liang JF 2010 | Kidney deficiency and blood stasis syndrome | *Bushenyizhi granule* | *Polygala tenuifolia 6g, Acorus tatarinowii 6g, prepared rehmannia root 10g, Morinda officinalis 10g, Cistanche deserticola 10g, longspur epimedium 10g, dendrobe 10g, Ophiopogon japonicus 10g, Poria cocos 10g, Salvia miltiorrhiza 10g, earthworm 10g, Cornus officinalis 6g, Schisandra chinensis 6g, Ligusticum wallichii 6g, leech 3g* | Yuanzhi 6g, Shichangpu 6g, Shudihuang 10g, Bjitian 10g, Roucongrong 10g, Yinyanghuo 10g, Shihu 10g, Maidong 10g, Fuling 10g, Danshen 10g, Dilong 10g, Shanzhuyu 6g, Wuweizi 6g, Chuanxiong 6g, Shuizhi 3g |
| Chen Y 2008 | Kidney Deficiency and Marrow Depletion Syndrome | *Huo Nao Fang* | *Polygala tenuifolia, Acorus tatarinowii, prepared rehmannia root, Cornus officinalis, Ganoderma lucidum, Radix Polygoni Multiflori Preparata, the fruit of Chinese wolfberry, Cistanche deserticola, longspur epimedium, Salvia miltiorrhiza, earthworm, Rhizoma Gastrodiae* | Yuanzhi, Shichangpu, Shudihuang, Shanzhuyu, Lingzhi, Zhishouwu, Gouqizi, Roucongrong, Yinyanghuo, Danshen, Dilong, Tianma |

Note: TCM: Traditional Chinese Medicine.
