# Supplementary material for: Polygala tenuifolia and Acorus tatarinowii in the treatment of Alzheimer’s disease: a systematic review and meta-analysis
Source: Front Pharmacol. 2024 Jan 12;14:1268000. doi: 10.3389/fphar.2023.1268000 (PMC10815298; doi:10.3389/fphar.2023.1268000)
Supplement: Supplementary file 2 [file Table2.docx]

**Supplementary Material 2. Search strategies**

| **Databases** | **Steps** | **Search String** |
| --- | --- | --- |
| PubMed | #1 | "Alzheimer Disease"[Mesh] |
|  | #2 | ((((("Alzheimer Disease"[Mesh]) OR (Alzheimer disease[Title/Abstract])) OR (Alzheimer*[Title/Abstract])) OR (dementia[Title/Abstract])) OR (AD[Title/Abstract])) OR (cogniti*[Title/Abstract]) |
|  | #3 | "Polygala"[Mesh] |
|  | #4 | ((((((((((("Polygala"[Mesh]) OR (Polygala)) OR (Yuanzhi)) OR (Yuan Zhi)) OR (Polygala tenuifolia)) OR (Polygala sibirica)) OR (Polygala senega)) OR (Seneca snakeroot)) OR (Milkwort)) OR (Polygala root)) OR (Polygalae Radix)) OR (Radix Polygalae) |
|  | #5 | "Acorus"[Mesh] |
|  | #6 | (((((((("Acorus"[Mesh]) OR (Acorus)) OR (Shichangpu)) OR (Shi Chang Pu)) OR (Acori Tatarinowii Rhizoma)) OR (Acorus gramineus)) OR (Acorus tatarinowii Schott)) OR (Acorus tatarinowii)) OR (Acorus calamus) |
|  | #7 | #1 OR #2 |
|  | #8 | #3 OR #4 |
|  | #9 | #5 OR #6 |
|  | #10 | #7 AND #8 AND #9 |
| Cochrane | #1 | MeSH descriptor: [Alzheimer Disease] explode all trees |
|  | #2 | (Alzheimer disease OR Alzheimer* OR dementia OR AD OR cogniti*):ti,ab,kw (Word variations have been searched) |
|  | #3 | #1 OR #2 |
|  | #4 | MeSH descriptor: [Polygala] explode all trees |
|  | #5 | (Polygala OR Yuanzhi OR Yuan Zhi OR Polygala tenuifolia OR Polygala sibirica OR Polygala senega OR Seneca snakeroot OR Milkwort OR Polygala root OR Polygalae Radix OR Radix Polygalae) (Word variations have been searched) |
|  | #6 | #4 OR #5 |
|  | #7 | MeSH descriptor: [Acorus] explode all trees |
|  | #8 | (Acorus OR Shichangpu OR Shi Chang Pu OR Acori Tatarinowii Rhizoma OR Acorus gramineus OR Acorus tatarinowii Schott OR Acorus tatarinowii OR Acorus calamus) (Word variations have been searched) |
|  | #9 | #7 OR #8 |
|  | #10 | #6 and #9 |
|  | #11 | #3 and #10 |
| Web of Science | #1 | TS=(Alzheimer disease OR Alzheimer* OR dementia OR AD OR cogniti*) |
|  | #2 | ALL=(Polygala OR Yuanzhi OR Yuan Zhi OR Polygala tenuifolia OR Polygala sibirica OR Polygala senega OR Seneca snakeroot OR Milkwort OR Polygala root OR Polygalae Radix OR Radix Polygalae) |
|  | #3 | ALL=(Acorus OR Shichangpu OR Shi Chang Pu OR Acori Tatarinowii Rhizoma OR Acorus gramineus OR Acorus tatarinowii Schott OR Acorus tatarinowii OR Acorus calamus) |
|  | #4 | #2 AND #3 |
|  | #5 | #1 AND #4 |
| Embase | #1 | 'alzheimer disease'/exp |
|  | #2 | 'alzheimer disease':ti,ab,kw OR 'alzheimer*':ti,ab,kw OR 'dementia':ti,ab,kw OR 'ad':ti,ab,kw OR 'cogniti*':ti,ab,kw |
|  | #3 | #1 OR #2 |
|  | #4 | 'polygalaceae'/exp |
|  | #5 | 'polygalaceae' OR 'polygala' OR 'yuanzhi' OR 'yuan zhi' OR 'polygala tenuifolia' OR 'polygala sibirica' OR 'polygala senega' OR 'seneca snakeroot' OR 'milkwort' OR 'polygala root' OR 'polygalae radix' OR 'radix polygalae' |
|  | #6 | #4 OR #5 |
|  | #7 | 'acorus'/exp |
|  | #8 | 'acorus' OR 'shichangpu' OR 'shi chang pu' OR 'acori tatarinowii rhizoma' OR 'acorus gramineus' OR 'acorus tatarinowii schott' OR 'acorus tatarinowii' OR 'acorus calamus' |
|  | #9 | #7 OR #8 |
|  | #10 | #6 AND #9 |
|  | #11 | #3 AND #10 |
| CNKI | #1 | SU='aercihaimo'+'chidai'+'laonianchidai'+'laonianxingchidai'+'shizhi'+'daibing' |
|  | #2 | TKA='aercihaimo'+'chidai'+'laonianchidai'+'laonianxingchidai'+'shizhi'+'daibing' |
|  | #3 | #1 OR #2 |
|  | #4 | FT='yuanzhi' |
|  | #5 | FT='shichangpu' |
|  | #6 | FT='suiji'+'RCT'+'duizhao' |
|  | #7 | 3 and 4 and 5 |
| Wanfang | #1 | Subject:(aercihaimo OR chidai OR laonianchidai OR laonianxingchidai OR shizhi OR daibing) or Title or Keywords:(aercihaimo OR chidai OR laonianchidai OR laonianxingchidai OR shizhi OR daibing) or Abstract:(aercihaimo OR chidai OR laonianchidai OR laonianxingchidai OR shizhi OR daibing) |
|  | #2 | All:(yuanzhi) and All:(shichangpu) |
|  | #3 | ALL:(suiji) or All:(RCT) or All:(duizhao) |
|  | #4 | #1 and #2 and #3 |
| VIP | #1 | Title or Keywords=aercihaimo OR chidai OR laonianchidai OR laonianxingchidai OR shizhi OR daibing |
|  | #2 | Any field=yuanzhi and shichangpu |
|  | #3 | Any field=suiji or RCT or duizhao |
|  | #4 | #1 and #2 and #3 |
| SinoMed | #1 | "aercihaimobing"[Unweighted: extended] |
|  | #2 | "aercihaimo"[Common Fields: intelligence] OR "chidai"[Common Fields: intelligence] OR "laonianchidai"[Common Fields: intelligence] OR "laonianxingchidai"[Common Fields: intelligence] OR "shizhi"[Common Fields: intelligence] OR "daibing"[Common Fields: intelligence] |
|  | #3 | "yuanzhi"[Unweighted: extended] |
|  | #4 | "yuanzhi"[All fields: intelligence] |
|  | #5 | "shichangpu"[Unweighted: extended] |
|  | #6 | "shichangpu"[All fields: intelligence] |
|  | #7 | "suijiduizhaoshiyan"[Unweighted: extended] |
|  | #8 | "suiji"[All fields: intelligence] OR "RCT"[All fields: intelligence] OR "duizhao"[All fields: intelligence] |
|  | #9 | (#2) OR (#1) |
|  | #10 | (#4) OR (#3) |
|  | #11 | (#6) OR (#5) |
|  | #12 | (#8) OR (#7) |
|  | #13 | (#11) AND (#10) |
|  | #14 | (#13) AND (#12) AND (#9) |
