# Supplementary material for: Polygala tenuifolia and Acorus tatarinowii in the treatment of Alzheimer’s disease: a systematic review and meta-analysis
Source: Front Pharmacol. 2024 Jan 12;14:1268000. doi: 10.3389/fphar.2023.1268000 (PMC10815298; doi:10.3389/fphar.2023.1268000)
Supplement: Supplementary file 4 [file Table5.docx]

# Supplementary Material 5

**Table 1 The effects of *PT and AT* on AD from 16 randomized controlled trials**

| **Outcome** | **No. of studies** | **No. of participants** | **I^2^, Model (REM/FEM)** | **Effect size** | **Quality of evidence (GRADE)** |
| --- | --- | --- | --- | --- | --- |
|  |  |  |  | **MD[95% CI]** |  |
| ***PT and AT* versus control** | | | | | |
| **MMSE** | 6 | 403 | 42%, REM | 0.33 [-0.64, 1.31] | Very low certainty. |
| **ADL** | 3 | 207 | 0%, FEM | -0.83 [-1.73, 0.08] | Very low certainty. |
| **ADAS-cog** | 3 | 207 | 0%, FEM | -0.66 [-1.69, 0.37] | Low certainty. |
| **TCM symptom score** | 2 | 140 | 0%, REM | -3.08 [-4.51, -1.64] | Very low certainty. |
| ***PT and AT* plus control versus control** | | | | | |
| **MMSE** | 10 | 697 | 86%, REM | 2.57 [1.44, 3.69] | Very low certainty. |
| **ADL** | 6 | 377 | 0%, FEM | -3.19 [-4.29, -2.09] | Low certainty. |
| **ADAS-cog** | 4 | 295 | 0%, FEM | -2.09 [-3.07, -1.10] | Moderate certainty. |
| **TCM symptom score** | 7 | 461 | 80%, REM | -4.32 [-5.89, -2.75] | Low certainty. |

Note: PT: *Polygala tenuifolia*; AT: *Acorus tatarinowii*; MD: mean difference; CI: confidence interval; No: number; REM: random-effects model; FEM: fixed-effects model; MMSE: Mini-mental state examination; ADL: Activities of daily living; ADAS-cog: AD assessment scale-cognitive subscale; TCM: Traditional Chinese Medicine.

**Table 2 The results of the subgroup analysis**

| **Outcome** | **Subgroup** | **No. of studies** | **No. of participants** | **I^2^, Model (REM/FEM)** | **Effect size** | **Quality of evidence (GRADE)** |
| --- | --- | --- | --- | --- | --- | --- |
|  | **Treatment durations** |  |  |  | **MD[95% CI]** |  |
| ***PT and AT* versus control** | | | | | | |
| **MMSE** | 3 m | 4 | 282 | 0%, FEM | -0.28 [-0.98, 0.43] | Very low certainty. |
|  | 6 m | 2 | 124 | 29% , FEM | 1.72 [0.01, 3.42] | Low certainty. |
| **ADL** | 3 m | 2 | 123 | 34%, FEM | -0.11 [-0.47, 0.24] | Very low certainty. |
|  | 6 m | 1 | 84 | 18%, FEM | -0.39 [-0.82, 0.05] | Low certainty. |
| **ADAS-cog** | 3 m | 2 | 123 | 0%, FEM | -0.80 [-2.17, 0.58] | Low certainty. |
|  | 6 m | 1 | 84 | 0%, FEM | -0.49 [-2.05, 1.07] | Very low certainty. |
| ***PT and AT* plus control versus control** | | | | | | |
| **MMSE** | 3 m | 5 | 338 | 92%, REM | 2.55 [0.96, 4.14] | Very low certainty. |
|  | 4 m | 1 | 30 | NA | 3.33 [0.19, 6.47] | Low certainty. |
|  | 6 m | 4 | 329 | 49% , REM | 2.27 [0.81, 3.74] | Low certainty. |
| **ADL** | 3 m | 2 | 122 | 0%, REM | -0.26 [-0.62, 0.10] | Very low certainty. |
|  | 4 m | 1 | 30 | NA | -0.88 [-1.63, -0.13] | Low certainty. |
|  | 6 m | 3 | 225 | 71%, REM | -0.61 [-1.12, -0.09] | Low certainty. |
| **ADAS-cog** | 3 m | 1 | 70 | NA | -2.40 [-4.38, -0.42] | Low certainty. |
|  | 6 m | 3 | 225 | 0%, FEM | -1.98 [-3.12, -0.84] | Low certainty. |
| **TCM symptom score** | 3 m | 3 | 206 | 71%, REM | -4.38 [-6.16, -2.60] | Very low certainty. |
|  | 4 m | 1 | 30 | NA | -8.00 [-15.46, -0.54] | Low certainty. |
|  | 6 m | 3 | 225 | 91%, REM | -4.03 [-7.36, -0.69] | Low certainty. |

Note: PT: *Polygala tenuifolia*; AT: *Acorus tatarinowii*; MD: mean difference; CI: confidence interval; No: number; REM: random-effects model; FEM: fixed-effects model; MMSE: Mini-mental state examination; ADL: Activities of daily living; ADAS-cog: AD assessment scale-cognitive subscale; TCM: Traditional Chinese Medicine.

**Supplemental Table 3** Sensitivity analysis of MMSE (PT and AT vs control)

| **Study omitted** | **Estimate** | **[95% Conf. Interval]** | |
| --- | --- | --- | --- |
| Yang L 2022 | .19078858 | -.04388272 | .42545989 |
| Yang F 2020 | .08075415 | -.217437 | .37894532 |
| Yang XC 2018 | .06917971 | -.21157546 | .34993491 |
| Zhang L 2018 | .15732327 | -.11881626 | .43346283 |
| Li XW 2017 | .11568873 | -.18517289 | .41655034 |
| Chen Y 2008 | .02769743 | -.18764517 | .24304003 |
| Combined | .10356305 | -.14205215 | .34917826 |

Effect sizes were pooled using random-effects mode

**Supplemental Table 4** Publication bias of MMSE (PT and AT vs control)

| Egger's test | | | | | | |
| --- | --- | --- | --- | --- | --- | --- |
| Std_Eff | Coef. | Std.Err | t | P>\|t\| | [95% Conf.Interval] | |
| slope | -1.221809 | .6676362 | -1.83 | 0.141 | -3.075464 | .6318463 |
| bias | 5.401158 | 2.730618 | 1.98 | **0.119** | -2.180252 | 12.98257 |

**Supplemental Table 5** Sensitivity analysis of MMSE (PT and AT plus control vs control)

| **Study omitted** | **Estimate** | **[95% Conf. Interval]** | |
| --- | --- | --- | --- |
| Wang SL 2022 | .97242093 | .44726673 | 1.4975752 |
| Guan XJ 2022 | .97052962 | .44702691 | 1.4940323 |
| Wang P 2021 | .67984623 | .38541859 | .97427392 |
| Gu YL 2019 | 1.0051471 | .49816024 | 1.5121341 |
| Guzainuer 2019 | .92898452 | .40705574 | 1.4509133 |
| Ling DX 2018 | .97329479 | .43546689 | 1.5111227 |
| Zheng R 2017 | .8045392 | .3478767 | 1.2612017 |
| Lin YQ 2017 | .98004663 | .46130875 | 1.4987845 |
| Peng XM 2014 | .94119942 | .43315378 | 1.449245 |
| Liang JF 2010 | .97849959 | .44442481 | 1.5125743 |
| Combined | .92339004 | .45379376 | 1.3929863 |

Effect sizes were pooled using random-effects mode

**Supplemental Table 6** Publication bias of MMSE (PT and AT plus control vs control)

| Egger's test | | | | | | |
| --- | --- | --- | --- | --- | --- | --- |
| Std_Eff | Coef. | Std.Err | t | P>\|t\| | [95% Conf.Interval] | |
| slope | -1.468106 | 1.0758 | -1.36 | 0.210 | -3.948906 | 1.012693 |
| bias | 8.883197 | 4.181299 | 2.12 | **0.066** | -.7588956 | 18.52529 |
