# Supplementary figures and images for: Polygala tenuifolia and Acorus tatarinowii in the treatment of Alzheimer’s disease: a systematic review and meta-analysis
Source: Front Pharmacol. 2024 Jan 12;14:1268000. doi: 10.3389/fphar.2023.1268000 (PMC10815298; doi:10.3389/fphar.2023.1268000)

**Supplementary Data 2. Risk of bias summary**


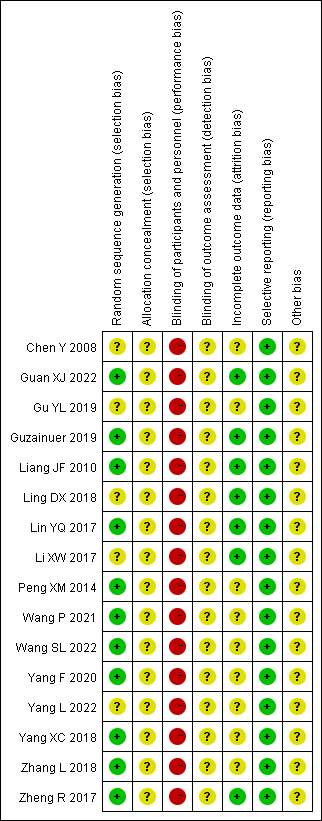


**Supplemental Fig. 1.** Risk of bias summary.

Supplement: Supplementary file 5 [file Table3.docx]
