# Supplementary material for: Polygala tenuifolia and Acorus tatarinowii in the treatment of Alzheimer’s disease: a systematic review and meta-analysis
Source: Front Pharmacol. 2024 Jan 12;14:1268000. doi: 10.3389/fphar.2023.1268000 (PMC10815298; doi:10.3389/fphar.2023.1268000)
Supplement: Supplementary file 6 [file Table4.docx]

**Supplementary Material 4. Forest plots of the effects of *Polygala Tenuifolia* and *Acorus Tatarinowii* in the Treatment of Alzheimer's Disease**


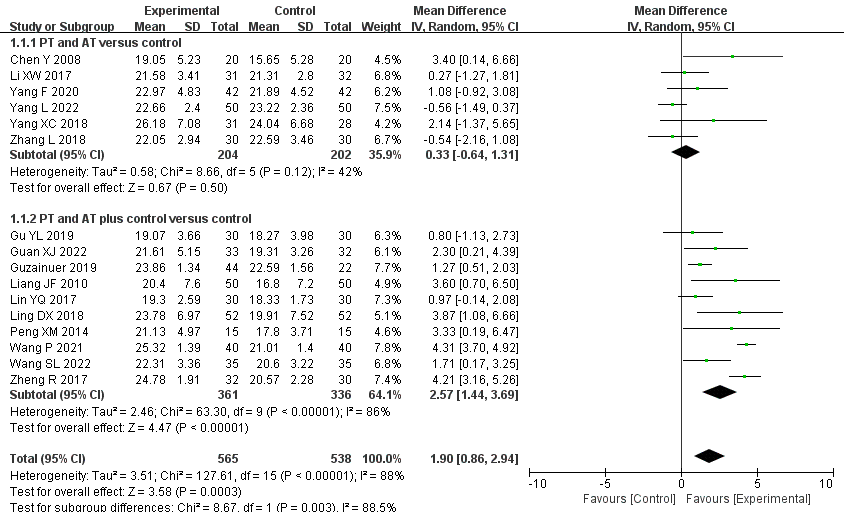


**Supplemental Fig. 1.** Forest plot of the effect of PT and AT on MMSE.


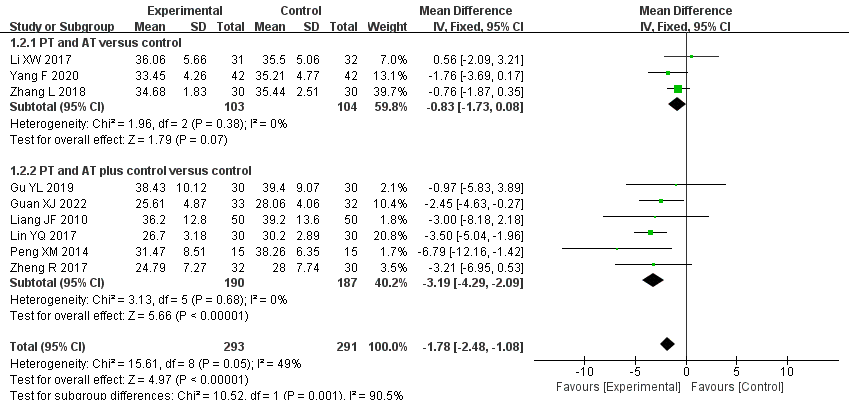


**Supplemental Fig. 2.** Forest plot of the effect of PT and AT on ADL.


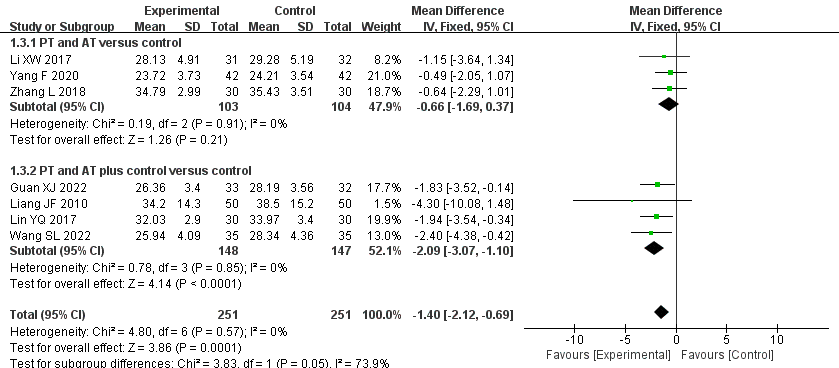


**Supplemental Fig. 3.** Forest plot of the effect of PT and AT on ADAS-cog.


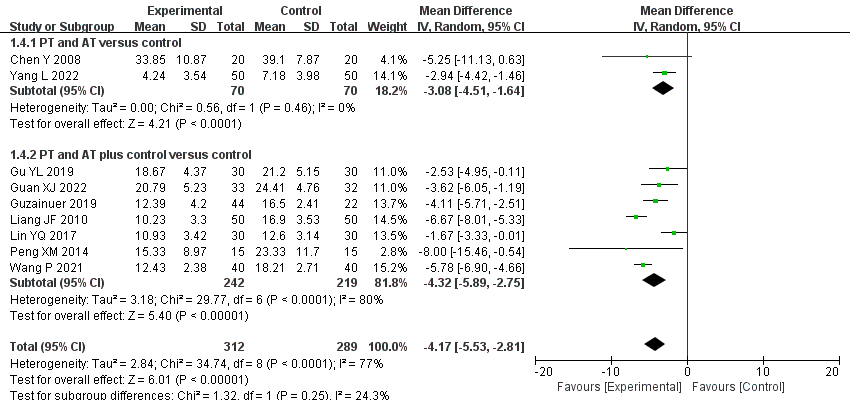


**Supplemental Fig. 4.** Forest plot of the effect of PT and AT on TCM symptom score.


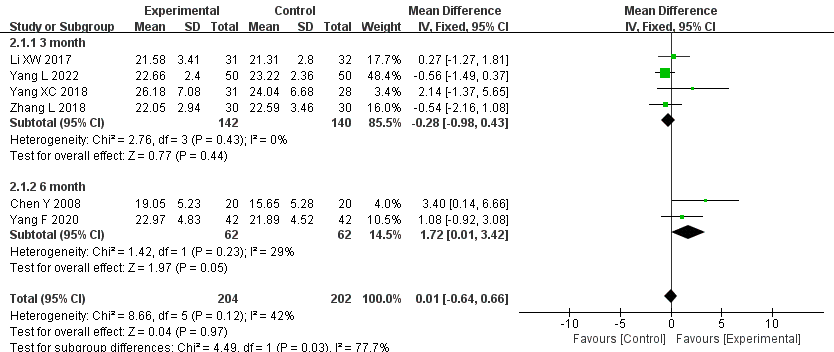


**Supplemental Fig. 5.** Forest plot of subgroup analysis of the effect of PT and AT on MMSE compared with control. (PT and AT versus control)


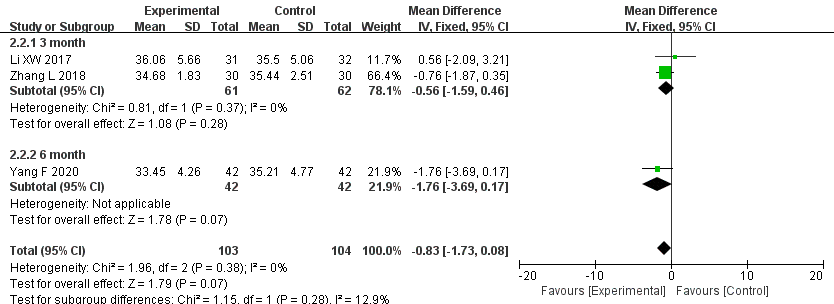


**Supplemental Fig. 6.** Forest plot of subgroup analysis of the effect of PT and AT on ADL compared with control. (PT and AT versus control)


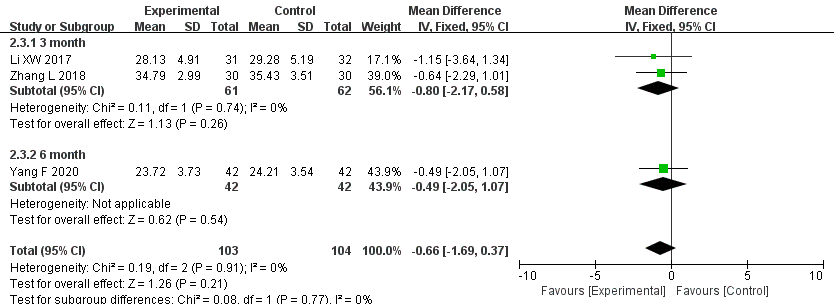


**Supplemental Fig. 7.** Forest plot of subgroup analysis of the effect of PT and AT on ADAS-cog compared with control. (PT and AT versus control)


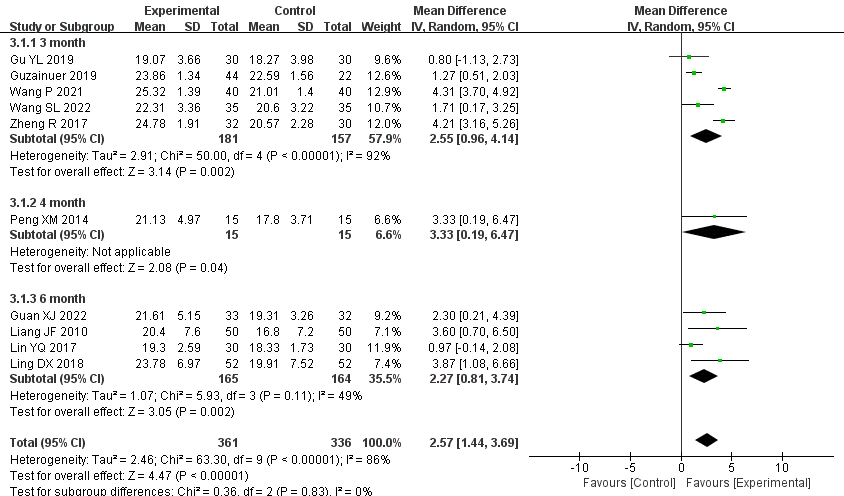


**Supplemental Fig. 8.** Forest plot of subgroup analysis of the effect of PT and AT on MMSE compared with control. (PT and AT plus control versus control)


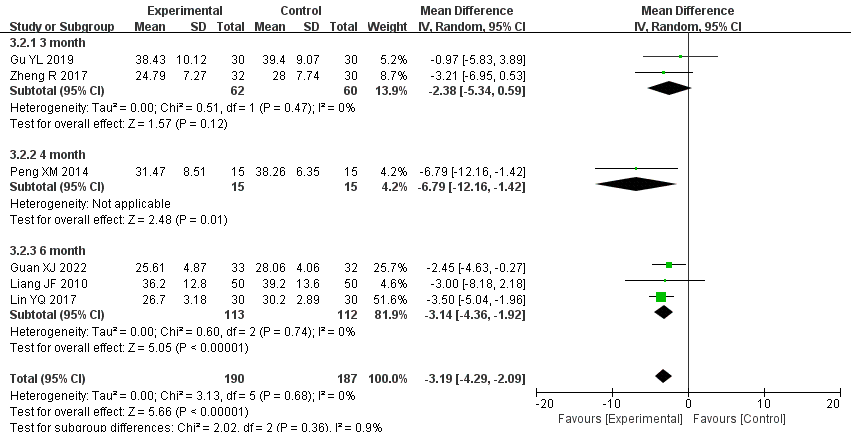


**Supplemental Fig. 9.** Forest plot of subgroup analysis of the effect of PT and AT on ADL compared with control. (PT and AT plus control versus control)


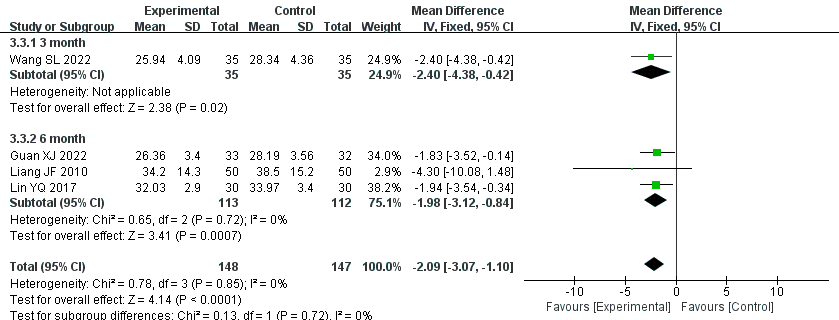


**Supplemental Fig. 10.** Forest plot of subgroup analysis of the effect of PT and AT on ADAS-cog compared with control. (PT and AT plus control versus control)


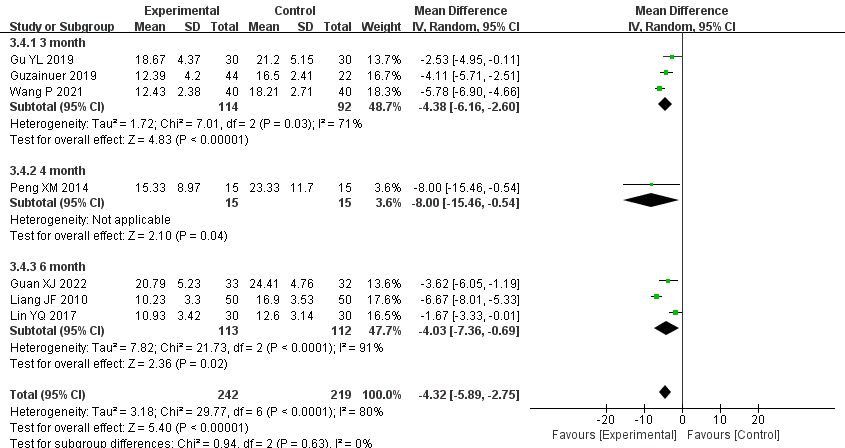


**Supplemental Fig. 11.** Forest plot of subgroup analysis of the effect of PT and AT on TCM symptom score compared with control. (PT and AT plus control versus control)


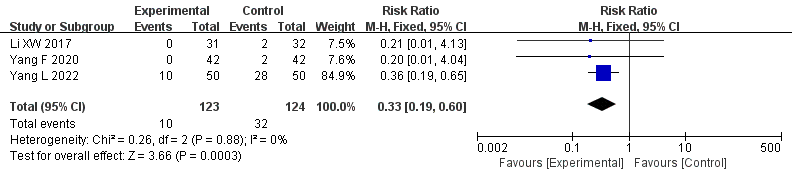


**Supplemental Fig. 12.** Forest plot of the effect of PT and AT on Adverse Reaction compared with control. (PT and AT versus control)


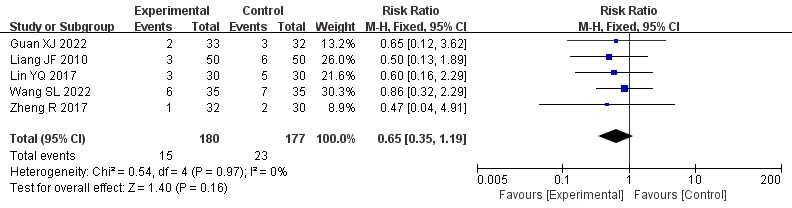


**Supplemental Fig. 13.** Forest plot of the effect of PT and AT on Adverse Reaction compared with control. (PT and AT plus control versus control)


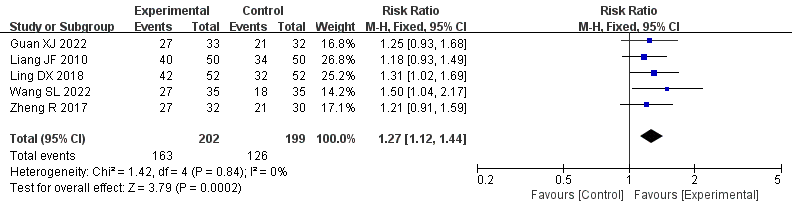


**Supplemental Fig. 14.** Forest plot of the effect of PT and AT on Clinical efficacy compared with control. (PT and AT plus control versus control)
