# Supplementary material for: Polygala tenuifolia and Acorus tatarinowii in the treatment of Alzheimer’s disease: a systematic review and meta-analysis
Source: Front Pharmacol. 2024 Jan 12;14:1268000. doi: 10.3389/fphar.2023.1268000 (PMC10815298; doi:10.3389/fphar.2023.1268000)
Supplement: Supplementary file 7 [file Table6.docx]

**Supplementary Material 6**

**Table 1. Results of evidence quality of outcomes.**

| **Outcome** | **No. of studies** | **No. of participants** | **Risk of bias** | **Inconsistency** | **Indirectness** | **Imprecisions** | **Publication bias** | **Quality of evidence (GRADE)** |
| --- | --- | --- | --- | --- | --- | --- | --- | --- |
| ***PT and AT* versus control** | | | | | | | | |
| **MMSE** | 6 | 406 | Serious ^a^ | Serious ^c^ | No serious | Serious ^e^ | Undetected | Very low certainty. |
| **ADL** | 3 | 207 | Serious ^a^ | No serious | No serious | Very serious ^b^ | Undetected | Very low certainty. |
| **ADAS-cog** | 3 | 207 | Serious ^a^ | No serious | No serious | Very serious ^b^ | Undetected | Very low certainty. |
| **TCM symptom score** | 2 | 140 | Serious ^a^ | No serious | No serious | Serious ^e^ | Undetected | Low certainty. |
| ***PT and AT* plus control versus control** | | | | | | | | |
| **MMSE** | 10 | 697 | Serious ^a^ | Very serious ^d^ | No serious | No serious | Undetected | Very low certainty. |
| **ADL** | 6 | 377 | Serious ^a^ | No serious | No serious | No serious | Undetected | Moderate certainty. |
| **ADAS-cog** | 4 | 295 | Serious ^a^ | No serious | No serious | Serious ^e^ | Undetected | Low certainty. |
| **TCM symptom score** | 7 | 461 | Serious ^a^ | Very serious ^d^ | No serious | No serious | Undetected | Very low certainty. |

**Notes**: PT: *Polygala tenuifolia*; AT: *Acorus tatarinowii*; No: number; MMSE: Mini-mental state examination; ADL: Activities of daily living; ADAS-cog: AD assessment scale-cognitive subscale; TCM: Traditional Chinese Medicine.

^a^ Large bias in randomization, allocation concealment and blinding in included studies;

^b^ The sample sizes of the included studies were small (< 300) and the confidence intervals were wide;

^c^ Represents heterogeneity (*I^2^*= 30% ‒ 75%);

^d^ Represents heterogeneity (*I^2^* > 75%);

^e^ Narrow or no overlap of confidence intervals;

^f^ Higher risk of publication bias.

**Table2. Results of evidence quality of outcomes in subgroup analysis.**

| **Outcome** | **Subgroup** | **No. of studies** | **No. of participants** | **Risk of bias** | **Inconsistency** | **Indirectness** | **Imprecisions** | **Publication bias** | **Quality of evidence (GRADE)** |
| --- | --- | --- | --- | --- | --- | --- | --- | --- | --- |
| ***PT and AT* versus control** | | | | | | | | | |
| **MMSE** | 3 m | 4 | 282 | Serious ^a^ | No serious | No serious | Very serious ^b^ | Undetected | Very low certainty. |
|  | 6 m | 2 | 124 | Serious ^a^ | No serious | No serious | Serious ^e^ | Undetected | Low certainty. |
| **ADL** | 3 m | 2 | 123 | Serious ^a^ | No serious | No serious | Very serious ^b^ | Undetected | Very low certainty. |
|  | 6 m | 1 | 84 | Serious ^a^ | No serious | No serious | Very serious ^b^ | Strongly suspected ^f^ | Very low certainty. |
| **ADAS-cog** | 3 m | 2 | 123 | Serious ^a^ | No serious | No serious | Very serious ^b^ | Undetected | Very low certainty. |
|  | 6 m | 1 | 84 | Serious ^a^ | No serious | No serious | Very serious ^b^ | Strongly suspected ^f^ | Very low certainty. |
| ***PT and AT* plus control versus control** | | | | | | | | | |
| **MMSE** | 3 m | 5 | 338 | Serious ^a^ | Very serious ^d^ | No serious | Serious ^e^ | Undetected | Very low certainty. |
|  | 4 m | 1 | 30 | Serious ^a^ | No serious | No serious | Serious ^e^ | Strongly suspected ^f^ | Very low certainty. |
|  | 6 m | 4 | 329 | Serious ^a^ | Serious ^c^ | No serious | No serious | Undetected | Low certainty. |
| **ADL** | 3 m | 2 | 122 | Serious ^a^ | No serious | No serious | Very serious ^b^ | Undetected | Very low certainty. |
|  | 4 m | 1 | 30 | Serious ^a^ | No serious | No serious | Serious ^e^ | Strongly suspected ^f^ | Very low certainty. |
|  | 6 m | 3 | 225 | Serious ^a^ | Very serious ^d^ | No serious | Serious ^e^ | Undetected | Low certainty. |
| **ADAS-cog** | 3 m | 1 | 70 | Serious ^a^ | No serious | No serious | Serious ^e^ | Strongly suspected ^f^ | Very low certainty. |
|  | 6 m | 3 | 225 | Serious ^a^ | No serious | No serious | Serious ^e^ | Undetected | Low certainty. |
| **TCM symptom score** | 3 m | 3 | 206 | Serious ^a^ | Serious ^c^ | No serious | Serious ^e^ | Undetected | Very low certainty. |
|  | 4 m | 1 | 30 | Serious ^a^ | No serious | No serious | Serious ^e^ | Strongly suspected ^f^ | Very low certainty. |
|  | 6 m | 3 | 225 | Serious ^a^ | Very serious ^d^ | No serious | Serious ^e^ | Undetected | Very low certainty. |

**Notes**: PT: *Polygala tenuifolia*; AT: *Acorus tatarinowii*; No: number; MMSE: Mini-mental state examination; ADL: Activities of daily living; ADAS-cog: AD assessment scale-cognitive subscale; TCM: Traditional Chinese Medicine.

^a^ Large bias in randomization, allocation concealment and blinding in included studies;

^b^ The sample sizes of the included studies were small (< 300) and the confidence intervals were wide;

^c^ Represents heterogeneity (*I^2^*= 30% ‒ 75%);

^d^ Represents heterogeneity (*I^2^* > 75%);

^e^ Narrow or no overlap of confidence intervals;

^f^ Higher risk of publication bias.


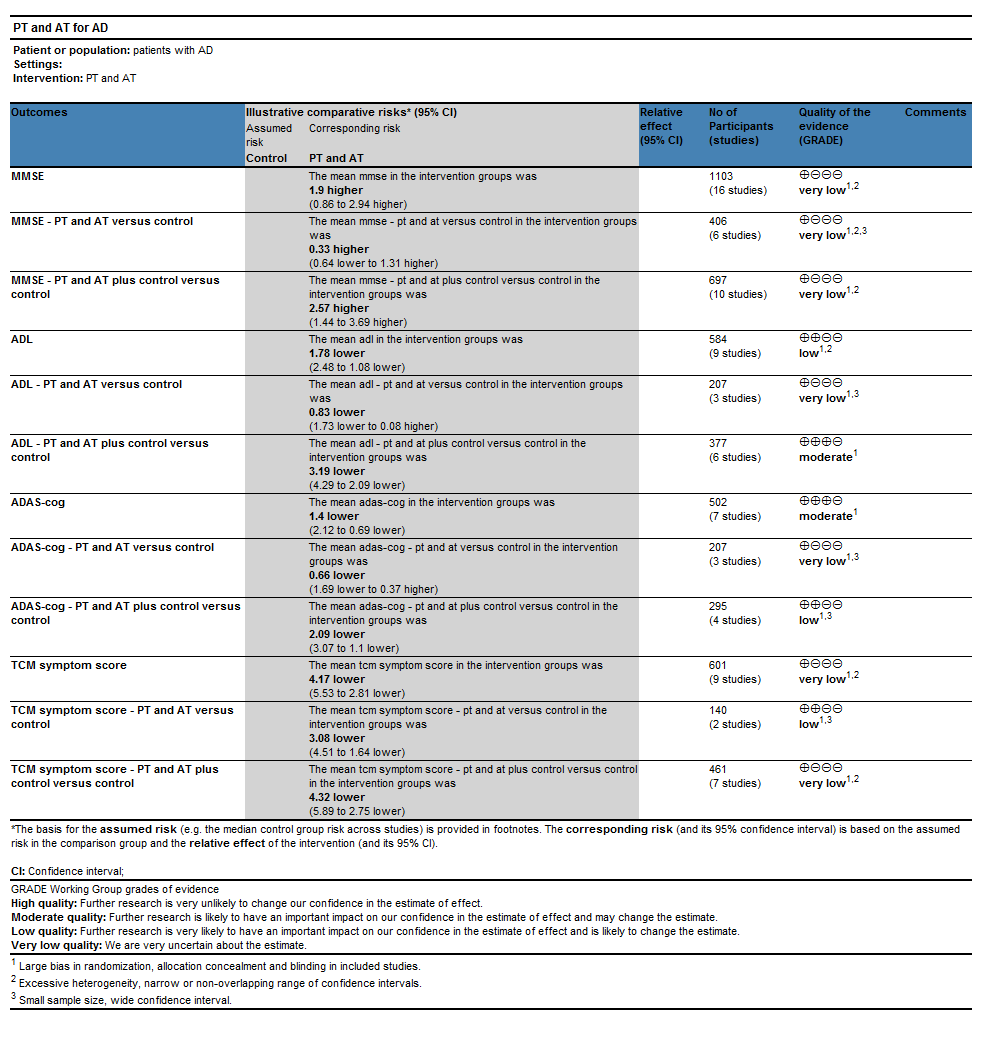


**Figure. 1** Results of evidence quality evaluation of GRADE for PT and AT

**Notes**: PT: *Polygala tenuifolia*; AT: *Acorus tatarinowii*; MMSE: Mini-mental state examination; ADL: Activities of daily living; ADAS-cog: AD assessment scale-cognitive subscale; TCM: Traditional Chinese Medicine.


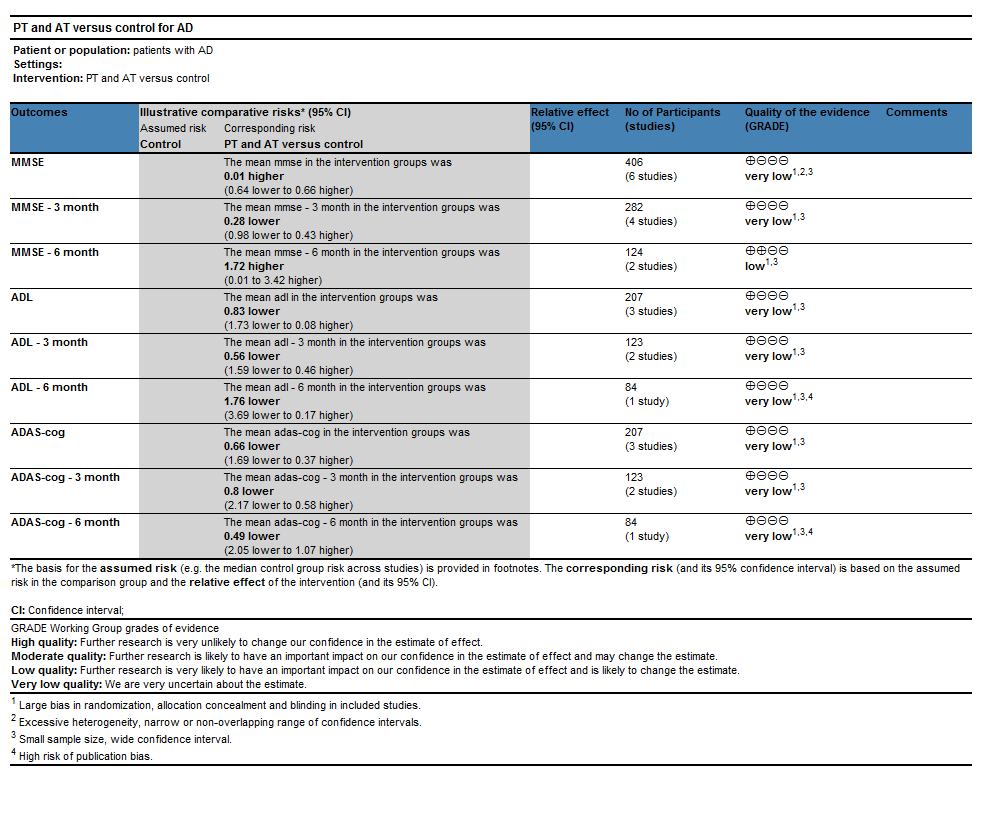


**Figure. 2** Results of evidence quality of outcomes based on treatment duration in subgroup analysis (PT and AT vs control).

**Notes**: PT: *Polygala tenuifolia*; AT: *Acorus tatarinowii*; MMSE: Mini-mental state examination; ADL: Activities of daily living; ADAS-cog: AD assessment scale-cognitive subscale; TCM: Traditional Chinese Medicine.


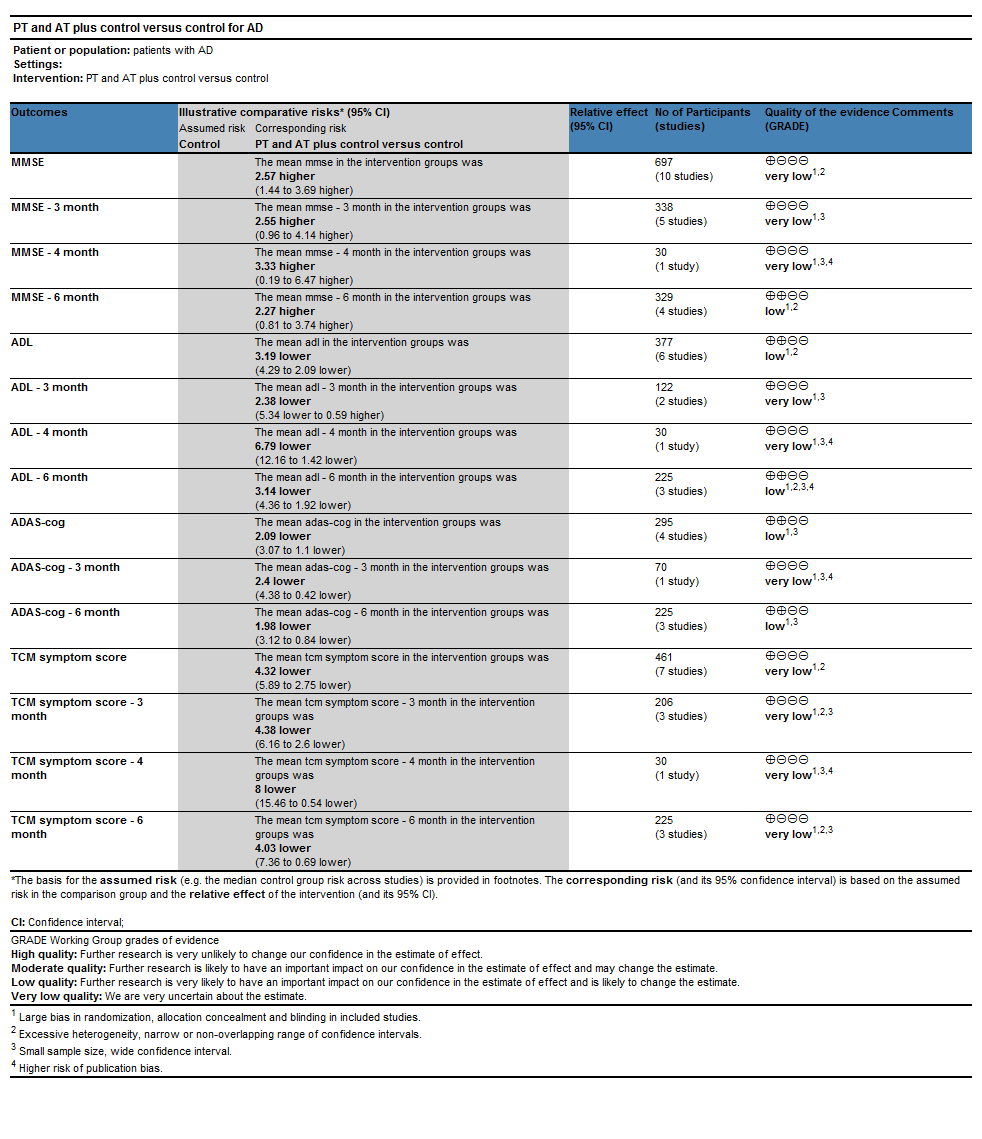


**Figure. 3** Results of evidence quality of outcomes based on treatment duration in subgroup analysis (PT and AT plus control vs control).

**Notes**: PT: *Polygala tenuifolia*; AT: *Acorus tatarinowii*; MMSE: Mini-mental state examination; ADL: Activities of daily living; ADAS-cog: AD assessment scale-cognitive subscale; TCM: Traditional Chinese Medicine.
